# A multi-institutional comparison of retrospective deformable dose accumulation for online adaptive magnetic resonance-guided radiotherapy

**DOI:** 10.1016/j.phro.2024.100588

**Published:** 2024-05-17

**Authors:** Martina Murr, Uffe Bernchou, Edyta Bubula-Rehm, Mark Ruschin, Parisa Sadeghi, Peter Voet, Jeff D Winter, Jinzhong Yang, Eyesha Younus, Cornel Zachiu, Yao Zhao, Hualiang Zhong, Daniela Thorwarth

**Affiliations:** a Section for Biomedical Physics, Department of Radiation Oncology, University of Tübingen, Germany; b Department of Clinical Research, University of Southern Denmark, Odense, Denmark; c Laboratory of Radiation Physics, Odense University Hospital, Denmark; d Elekta AB, Stockholm, Sweden; e Department of Radiation Oncology, Odette Cancer Centre, Sunnybrook Health Sciences Centre, University of Toronto, Toronto, Ontario, Canada; f Radiation Medicine Program, Princess Margaret Cancer Centre, University Health Network, Toronto, Ontario, Canada; g Department of Radiation Physics, the University of Texas MD Anderson Cancer Center, Houston, TX, USA; h University Medical Centre Utrecht, Department of Radiotherapy, 3584 CX Utrecht, the Netherlands; i Department of Radiation Oncology, Medical College of Wisconsin, Milwaukee, WI, USA; j Department of Radiation Oncology, Mayo Clinic, 200 First Street SW, Rochester, MN, 55905, USA

**Keywords:** Deformable dose accumulation (DDA), Deformable image registration (DIR), Multi-institutional analysis, Online MR-guided radiotherapy (MRgRT)

## Abstract

**Background and Purpose:**

Application of different deformable dose accumulation (DDA) solutions makes institutional comparisons after online-adaptive magnetic resonance-guided radiotherapy (OA-MRgRT) challenging. The aim of this multi-institutional study was to analyze accuracy and agreement of DDA-implementations in OA-MRgRT.

**Material and Methods:**

One gold standard (GS) case deformed with a biomechanical-model and five clinical cases consisting of prostate (2x), cervix, liver, and lymph node cancer, treated with OA-MRgRT, were analyzed. Six centers conducted DDA using institutional implementations. Deformable image registration (DIR) and DDA results were compared using the contour metrics Dice Similarity Coefficient (DSC), surface-DSC, Hausdorff-distance (HD95%), and accumulated dose-volume histograms (DVHs) analyzed via intraclass correlation coefficient (ICC) and clinical dosimetric criteria (CDC).

**Results:**

For the GS, median DDA errors ranged from 0.0 to 2.8 Gy across contours and implementations. DIR of clinical cases resulted in DSC > 0.8 for up to 81.3% of contours and a variability of surface-DSC values depending on the implementation. Maximum HD95%=73.3 mm was found for duodenum in the liver case. Although DVH ICC > 0.90 was found after DDA for all but two contours, relevant absolute CDC differences were observed in clinical cases: Prostate I/II showed maximum differences in bladder V28Gy (10.2/7.6%), while for cervix, liver, and lymph node the highest differences were found for rectum D2cm^3^ (2.8 Gy), duodenum Dmax (7.1 Gy), and rectum D0.5cm^3^ (4.6 Gy).

**Conclusion:**

Overall, high agreement was found between the different DIR and DDA implementations. Case- and algorithm-dependent differences were observed, leading to potentially clinically relevant results. Larger studies are needed to define future DDA-guidelines.

## Highlights

• A multi-center study was performed comparing six different deformable image registration and dose accumulation implementations using a gold standard case with known deformations and five clinical cases which had been treated with magnetic resonance-guided radiotherapy (MRgRT).
• Deformable image registration (DIR) of clinical cases resulted in Dice Similarity Coefficient (DSC) > 0.8 for up to 81.3% of contours and a variability of surface-DSC values depending on the implementation.
• Dose volume histogram (DVH) related intraclass correlation coefficients (ICC) showed excellent reliability (>0.9) between different DVHs except for two, although considerable absolute differences in clinical dosimetric criteria were observed in the clinical cases.
• The algorithms used in this study showed a generally high level of agreement, but significant differences were found between the clinical cases and the algorithms, which may have clinical relevance.

## 1 Introduction

Online magnetic resonance-guided radiotherapy (MRgRT) enables optimal plan adaptation concerning the patient’s daily anatomy, allowing improved target coverage and organ at risk (OAR) sparing [1]. Deformable dose accumulation (DDA) is a method to evaluate doses from several treatment fractions deformed according to daily images to account for anatomical changes during treatment [2]. According to several recent studies, DDA promises precise adaption of delivered organ-specific doses to reduce OAR toxicity [3], [4], [5], more precisely determined dose tolerances [6], and/or additional target dose escalation [5]. However, DDA still requires investigation of dose mapping uncertainties and anatomical or contouring variations [7].

Deformable image registration (DIR) is performed as first step of DDA. Several DIR algorithms, categorized by matching criteria and deformation model, are available for clinical and research use, like intensity or hybrid methods and free-form deformations or diffusion/deformation models [8]. As recently published, underlying algorithms itself typically works differently in various anatomical regions [9], [10], [11], [12]. Additionally, different algorithms applied to one anatomical region can lead to different results [10], [13], and algorithm settings such as different configuration values or optimization methods may change registration results [14].

The deformation vector field (DVF) generated by DIR is applied to the corresponding 3D dose map in the second step of DDA. In literature, several resampling and interpolation approaches are discussed, especially direct dose mapping (DDM) and energy mass transfer (EMT) [[2], [15]]. DDA algorithms are currently being applied using various implementations across institutions, which may provide different results for the same problem, as several studies show [[16], [17]]. For MR-guided liver stereotactic body radiation (SBRT), Wahlstedt et al. [16] observed that DDA algorithms are highly patient- and fraction-dependent and recommend using numerous algorithms in tandem. Bosma et al. [17] found that in MRgRT of prostate cancer, EMT led to lower dose errors than DDM.

This multi-institutional investigation aimed to assess several different implementations for DDA utilizing the same datasets including a gold standard (GS) case deformed using a biomechanical model and five clinical cases. The goal was to identify differences in results based on the algorithms used.

## 2 Material and methods

The multicenter DDA study was conducted by a working group of the Elekta MR-Linac consortium involving six institutions with clinical expertise in MRgRT using the 1.5 T MR-Linac (Unity, Elekta AB, Sweden).

### 2.1 Data characteristics

Initially, DDA was conducted on a prostate case where deformations were created by biomechanical simulations. This process included loading clinical contours into finite element modeling software (FEBioStudio v1.7.1) [18], followed by assigning the physical attributes to each anatomical tissue [17]. Simulations of five clinically representative bladder and rectal fillings were used to actuate surrounding tissue and apply displacements and deformations to a T2-weighted MR image (MRI) (Supplementary Fig. A.1). For each simulated anatomical situation, RT plans were created by recalculating the dose on the new anatomy, resulting in five total dose distributions. The known underlying deformations enabled the calculation of a GS accumulated dose (GS-DDA) using DDM.

Subsequently, five clinical datasets of patients with different tumor entities were analyzed, including (I, II) two prostate, one (III) cervix, (IV) liver, and (V) lymph node cases, as detailed in Supplementary Table A.1 and Fig. A.2. Patients were treated at one participating institute with SBRT on the 1.5 T MR-Linac using the 'adapt-to-shape' (ATS) workflow [1]. Experienced radiation oncologists re-contoured the MRI for each fraction offline to address minor issues eventually caused by the tight timeline for online contouring. Subsequently, wall contours for rectum, colon, and duodenum, likewise for bladder and stomach, were created using negative margins of 3 and 4 mm to investigate DIR and DDA results in the hollow organs with homogeneous fillings. These fraction data sets, including MRI, re-delineated contours, and dose files, were shared using ProKnow D (Elekta AB, Sweden, V1.33.0). Clinical dosimetric criteria (CDC) used for plan approval were provided by the institution for each specific case (Supplementary Table A.2). Ethical approval and data sharing agreement existed for all clinical cases.

### 2.2 Deformable image registration, contour propagation, and dose accumulation

Each institute performed DIR using the software available at their institute. Two institutes (A, C) had a hybrid intensity/structure-based algorithm (Monaco ADMIRE Research, Elekta, Sweden) [19] or ANACONDA (RaySearch Laboratories AB, Sweden) [20]. Mixed/hybrid and contour-guided DIR algorithms [21] (MIM Software Inc., USA, versions 6.8.5/7.0.6) were used by institutes B and D, respectively. Institute E used an in-house intensity-based DIR algorithm [[22], [23]]. RTTracker (UMC, Utrecht, The Netherlands) [[24], [25]] was used by institute F. Supplementary A, including Table A3, describes each algorithm in more detail. Five institutions used contour-guided DIR, while one institution (E) did not (cf. Table 1). All MRIs were deformably registered to the first fraction. Each institution propagated all offline re-delineated contours of fractions 2–5 to fraction 1 using the resulting DVF.

**Table 1:** Overview of contours used for DIR guidance per institute (A-F) for the cases gold standard, (I) prostate 1, (II) prostate 2, (III) cervix, (IV) liver, (V) lymph node. Abbreviations; CTV: clinical target volume, GTV: gross tumor volume, CTV_HR_: high risk CTV, CTV_IR_: intermediate risk CTV, CTV_R_: right lymph node CTV, CTV_L_: left lymph node CTV.

Institute	A	B	C	D	E	F
**Gold Standard**	Bladder, femurs, rectum	CTV, bladder, rectum	CTV, bladder, rectum	CTV, bladder, rectum, sphincter	None	CTV, GTV, bladder, rectum
**(I) Prostate 1**						
**(II) Prostate 2**						
**(III) Cervix**	Bladder, rectum, sigmoid	CTV_HR_, bladder	Bladder, rectum, sigmoid	CTV_HR_, CTV_IR_, GTV, bladder, rectum, sigmoid	None	CTV_HR_, bladder, rectum
**(IV) Liver**	Colon, duodenum, kidneysmall bowel, spinal cord, stomach	GTV, liver	GTV, duodenum, liver, pancreas, stomach	GTV, colon, duodenum, kidney, pancreas, spinal cord, small bowel	None	GTV, duodenum, stomach
**(V) Lymph node**	Bladder, rectum, sigmoid	Rectum	CTV_L_, CTV_R_, bladder, rectum	Bladder, femurs, pelvis, rectum, sacrum	None	CTV_L_, CTV_R_, bladder, rectum

Dose mapping was conducted by applying the DVF to the dose file of each fraction. For dose resampling, two distinct methods were used: DDM [26] (A-E) and EMT [27] (F). The software solutions Slicer3D (V4.11) [28], Raystation (8B), MIM, an in-house developed solution, and RTTracker (V4.0) were used, respectively. No additional DIR/DDA requirements were made for the institutes.

### 2.3 Data analysis

Each institute’s accumulated dose maps for the GS case were compared with the known GS-DDA, calculating voxel-wise absolute differences within the target volume and OARs. Median (interquartile range, IQR) were compared, as a normal distribution could not be assumed. Contour propagation of all cases was evaluated by the Dice Similarity Coefficient (DSC) [29], surface DSC (SDSC) with 2 mm threshold [30] and 95% Hausdorff-distance (HD95%) [[31], [32]] using MATLAB (R2020b). Corresponding dose-volume-histograms (DVH) were generated to evaluate the accumulated doses. All relevant CDC derived from these DVHs were calculated and compared for all cases and institutions using ProKnow. Furthermore, the DVHs and CDCs for the wall structures were calculated.

The Kruskal-Wallis test was used to determine whether there were significantly different in the dose errors reported by the six institutes for the GS. The institutes were also compared pairwise via a Mann-Whitney test [33]. A repeated measures ANOVA was conducted to ascertain variances among the means of distinct algorithms, while a post-hoc test was employed to discern specific group disparities. These analyses were undertaken to evaluate significant differences across institutes concerning DSC and CDC metrics. The tests were conducted at a significance level of 5%.

Intraclass correlation coefficients (ICC) were computed to test the similarity of the different accumulated DVH curves, indicating the level of correlation and agreement between the DVHs determined using the various algorithms [34]. A high ICC indicates a high level of agreement between the DVHs, while a low ICC indicates a low level of agreement. ICC estimates and their 95% confidence intervals (CI) were computed using SPSS statistical package version 23 (SPSS Inc, Chicago, IL), using a single-rating, consistency, 2-way mixed-effects model [34].

## 3 Results

**Fig. 1:**
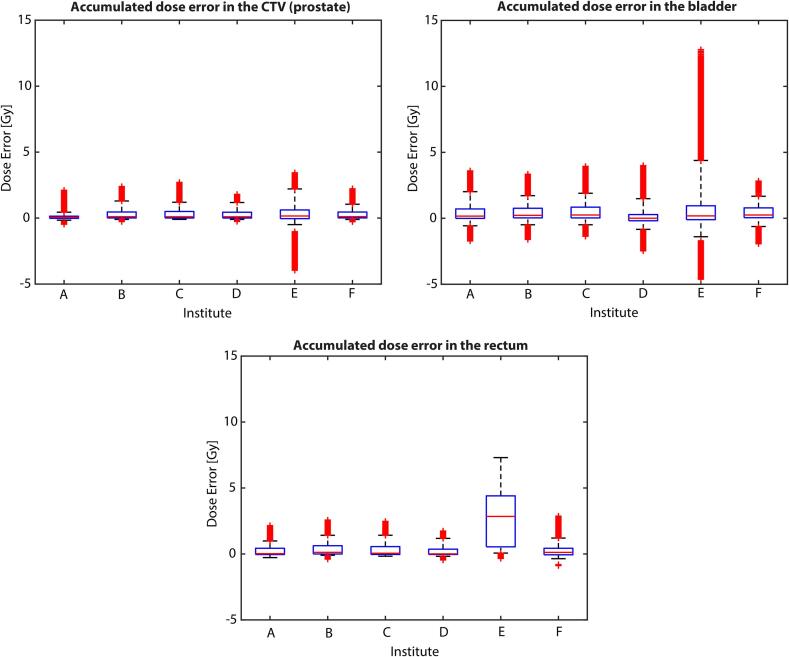
Statistical distribution of the accumulated dose errors for each participating institute. The illustrations are made individually for the bladder, prostate, and rectum. For each of the boxplots, the box limits correspond to the 25th and the 75th percentiles of the set, while the whiskers are the 5th and the 95th percentiles. The red line inside each box indicates the median of the set, with the red markers beyond each whisker showcasing the outliers. (For interpretation of the references to colour in this figure legend, the reader is referred to the web version of this article.)

For the GS, very good agreement was achieved by 5/6 institutes. Institute E reported higher median (IQR) dose accumulation errors for clinical target volume (CTV) and rectum, 0.2 (0.7) and 2.8 (3.7) Gy, respectively, compared to the other institutes. In contrast, the highest median errors were found for bladder by institutes C and F with 0.2 (0.8) and 0.2 (0.7) Gy, respectively, whereas institute E presented the highest IQR of 1.1 Gy. Fig. 1 presents the full statistical distribution of the voxel-based dose differences, with further details available in Supplementary Table B.1. The Kruskal-Wallis test rejected the null hypothesis for all three contours, indicating statistically significant institution differences, only institutes C and F demonstrated statistically insignificant differences within the CTV. Similar observations were obtained within the rectum for institutes A and D.

**Fig. 2:**
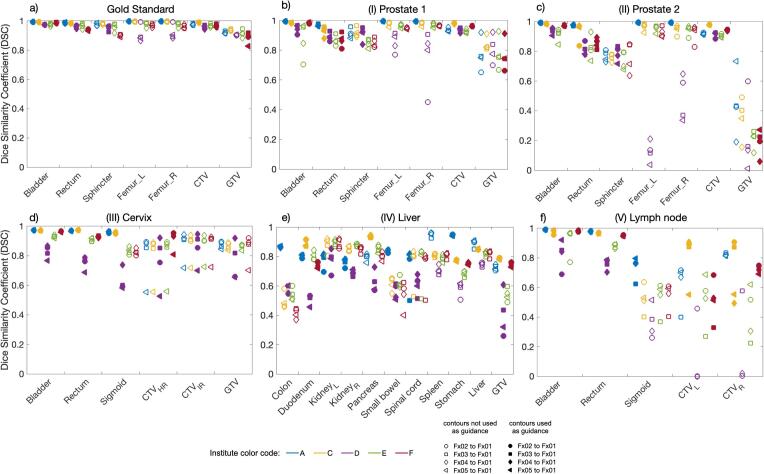
Illustration of the dice similarity coefficient (DSC) results for the cases gold standard (a), (I) prostate 1 (b), (II) prostate 2 (c), (III) cervix (d), (IV) liver (e), and (V) lymph node (f). Case-related deformed contours are listed on the x-axis. The results of the institutes (A, C, D, E, F) are shown in different colors, institute B did not provide deformed contours. The results of the different fractions are shown in different marker types. Abbreviations; CTV: clinical target volume, GTV: gross tumor volume, CTV_HR_: High risk clinical target volume, CTV_IR_: Intermediate risk clinical target volume, CTV_R_: right lymph node, CTV_L_: left lymph node.

Comparing DSC results per contour and institute for the GS and clinical cases, all DSC values were above 0.8 for GS, whereas a DSC > 0.8 was met in 81.3%, 75.7%, 34.6%, 64.6%, and 68.8% of contours by institutes A, C, D, E, and F for the clinical cases. Fig. 2 illustrates the DSCs for each patient. The ANOVA for repeated measures resulted in statistically non-significant DSC differences for GTV in case (I), and for sphincter and GTV in case (II). Similarly, in case (III), non-significant differences were found for CTV_HR_, CTV_IR_, and GTV, as well as for spinal cord in case (IV). Detailed DSC results for each case are summarized in Supplementary Tables B.2–B.7. The ANOVA and pairwise post-hoc test results are given in Supplementary Table B.8. The SDSC values for the GS case showed slight differences in the algorithm performances, with F consistently achieving higher SDSC values in most contours. Conversely, in the clinical cases, more pronounced disparities were noted in the SDSC values among different algorithms, notably with A, C, and E reaching higher values. For the GS, a maximum HD95% of 7.2 mm was found for femur_L_. In contrast, maximum HD95% of 73.3 mm was observed for duodenum in clinical case (IV), for details see Supplementary Tables B.2–B.7. The most challenging area seemed to be the left lymph node in case (V), where the target consisted of two independently moving lymph nodes. An in-depth motion analysis of the two lymph nodes is presented in Supplementary Fig. B.1.

**Fig. 3:**
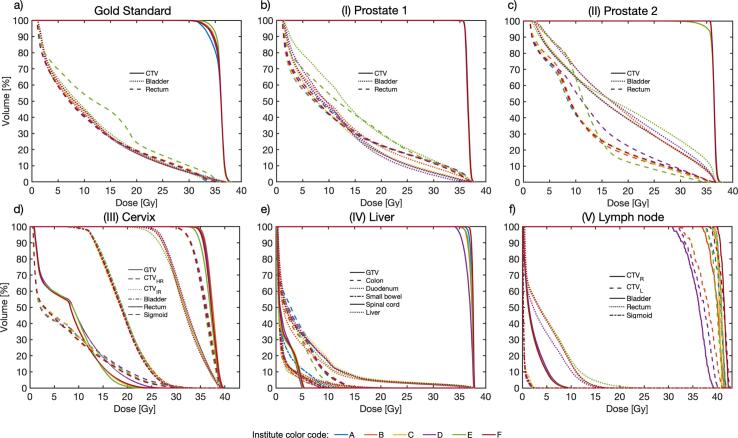
Results of the accumulated DVH per structure and institutes; for the cases gold standard (a), (I) prostate 1 (b), (II) prostate 2 (c), (III) cervix (d), (IV) liver (e) and (V) lymph node (f). The institutes (A-F) are presented in different colors, the case-related contours are presented in different line styles. Contours of fraction one were used for the respective DVH calculation.

**Fig. 4:**
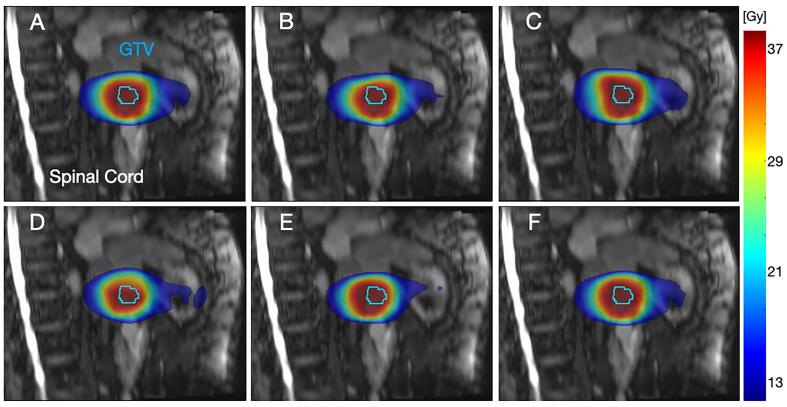
Sagittal plane view of institutional deformed dose accumulation (DDA) distribution results of the liver case (subfigures A-F are presenting the different institutes A-F). The gross tumor volume (GTV) contour of fraction one is presented in all images.

In Fig. 3, DDA DVHs for every case and contour are displayed. ICC > 0.90 indicated excellent correlation for all DVHs, except for CTV_L_ and CTV_R_ in case (V), with ICCs (95% CI) of 0.84 (0.84–0.85) and 0.72 (0.71–0.73), respectively. However, significant differences (ANOVA, p < 0.05) were found for all contour DVHs, except for CTV case (I), GTV and spinal cord case (IV), and bladder case (V). ICC, ANOVA and pairwise post-hoc test results are summarized in Supplementary Table B.9. Fig. 4 visualizes the different deformed accumulated dose distributions exemplarily for case (IV). Resulting DHVs of the deformed fraction doses are shown in Supplementary Fig. S2. The DVHs of the wall structures are shown in Supplementary Fig. S3.

The largest range of OAR CDC values in the GS was observed for rectum V32Gy with 4.1% [3.5%–7.6%]. In clinical cases (I) and (II), largest OAR CDC range was found for bladder V28Gy with 10.2% [5.9%–16.1%] and 7.6% [22.4%–30.0%], respectively. Similarly, the range of rectum D2cm^3^ in case (III) was 2.8 [16.5–19.3] Gy. For the duodenum, case (IV), differences in Dmax of up to 7.1 [16.6–23.7] Gy were observed. For case (V), the largest range of CDC values was found for rectum D0.5cm^3^ with 4.6 [14.1–18.7] Gy. CTV CDC showed largest variation for GS V34.3 Gy and case (V) D98% (CTV_R_ and CTV_L_) with 9.5% [86.3%-95.8%], 8.1 [31.9–40.0] Gy, and 6.5 [33.1–39.6] Gy, respectively. Details on CDC results are given in Supplementary Table B2, results of wall structures are summarized in Supplementary Table B10.

## 4 Discussion

In this study, DDA was performed on a GS with known deformations based on simulations using a biomechanical modelling, in addition to five distinct clinical cases. A total of six different DDA approaches were investigated, using various contour guiding methods and two different dose resampling techniques, DDM and EMT. In general, a high degree of agreement was found between the different DIR and DDA assessments. However, absolute differences of potentially clinically relevant magnitude were found depending on the clinical cases and algorithms.

The finite element simulation enabled the calculation of GS-DVF of both forward and backward transformations, which are nearly inverse to each other within the size of a voxel. GS-DVF enabled consistent mapping of image and dose from the first fraction to the others and vice versa, allowing accuracy evaluation of the different DDA solutions compared to the GS-DDA. It is crucial to note that while the simulated deformations show a high level of anatomical accuracy [17], they do not completely replicate the intricacy of the true deformations in the pelvis. Nevertheless, the DIR solution without contour guidance (E) resulted for CTV and rectum in notably higher DDA errors and for bladder in the highest IQR but showed similar contour propagation performance to other solutions. This might be due to algorithm regularization and poor anatomical contrast inside the contour boundaries, causing large uncertainty in the estimated deformations and in turn leading to dose wrapping/accumulation errors. This highlights the need for DIR and DDA QA metrics that detect errors not just in high-contrast image areas like organ boundaries but also in near-isointense areas often seen in soft tissue boundaries [[35], [36]]. Statistical testing has largely shown significant differences in dose accumulation accuracy among the institutions. On the other hand, it is worth noting that such differences are often between 1 and 2% of the maximum prescribed dose and thus their clinical relevance depends on the specific requirements.

By nature, for the clinical cases no DDA ground truth was available. A DVF and dose distribution analysis would provide a precise estimation of deformation results [7]. Unfortunately, this analysis was not possible due to export and reading issues and requires additional investigation. Alternatively, the percentages of contours yielding DSC > 0.8 were reported per algorithm, in addition to SDSC and HD95% were calculated. Nevertheless, it is worth noting that DSC may have restricted sensitivity to local registration errors, particularly for volumes of varying sizes. The clinical cases presented more variations in SDSC values among different algorithms as in the GS. Algorithms A, C, and E presented higher SDSC values compared to others in the clinical cases, indicating their relatively better performance in clinical scenarios.

The largest HD95% of 73.3 mm was found for duodenum in case (IV) which was not clinically concerning as this contour region was far from the high- or near-high-dose area. Nevertheless, this indicates remaining registration uncertainties underlining the need for appropriate registration strategies depending on clinical priorities. In contrast, for the CTVs of case (V), a maximum HD95% of 4.3 mm was reported, showing more robust results in high-dose regions.

In our study, offline re-delineations were used for DIR and DDA analysis. Instead of using propagated contours used for DIR guidance, re-delineation of deformed images and subsequent comparison to reference contours may be an alternative approach for investigating DIR quality. Unfortunately, such contours were not available for our analysis. However, it should be noted that the contours from the first fraction were used as guidance. Consequently, observed DSC reduction may be caused by registration errors or delineation variation. We acknowledge that manual contouring is subject to inter- and intra-observer variability [[37], [38]]. However, this may also be observed in clinical practice and thus requires thorough retrospective analysis. In contrast, re-delineation in online adaptive RT must be fast and accurate to satisfy the precision standards for DDA analysis. Automated deep-learning algorithms are currently developed for fast and robust auto-contouring [[39], [40], [41]]. These tools may soon be available for online adaptive RT, enabling contour-guidance for online DIR.

Analysis of DSC, SDSC and HD95% showed that the DIR algorithm relaying on contours only performed poorest overall. This algorithm generally works well in regions near contours but deteriorates significantly in more distant regions, such that the distance between the assessed OAR and the guiding contours can lead to significantly decreased registration results. Furthermore, the accuracy of this algorithm seems to decrease if more contours are used for guidance, most likely due to overfitting and increasing model complexity. However, the study of Wahlstadt et al. [16] revealed best performance for contour-only and hybrid algorithms in terms of DSC and HD95%, while the hybrid and intensity-based algorithms performed best for image similarity metrics in liver. In our study, consistent DIR results were found for hybrid intensity/structure-based algorithms, suggesting this might be well suited for clinical DDA. Similar results were found by Bosma et al. [37], who showed that contour-guidance significantly increased registration accuracy. However, due to the low number of investigated approaches no conclusion about the optimal registration strategy can be derived from this study.

An alternative comparison of DDA strategies was performed by comparing resulting DVHs of the different strategies using ICC and ANOVA. Even though ICCs indicated high correlation of DVHs, absolute differences in CDC were observed. Although the CDC presents a single DVH point, it may be of clinical relevance and observed differences require further clinical evaluation as they might impact toxicity and target coverage after online adaptive RT DDA. Low ICCs were observed for left and right CTV in case (V), due to inter-fraction motion of the lymph nodes [38], their very small volumes [42], and image resolution [43]. As an alternative approach, separate rigid registration of each lymph node with dose summation might be considered. Nevertheless, to estimate potential toxicity in the genitourinary system, DDA should be preferred.

The evaluation of the largest differences in the considered OAR CDC showed that the algorithm without contour-guidance (E) yielded minimum or maximum values, even though demonstrating better DSC, SDSC and HD95% results compared to the contour-only algorithm (D). This might be related to registration uncertainties near the high-dose gradient, i.e. a larger distance between points in high-dose areas results in small dose differences as opposed to points in gradient areas near the high-dose [7].

Regarding DDM and EMT, no valid statement can be made, as there was not enough variation in dose mapping approaches in this study. Furthermore, the decision about EMT or DDM can become rather complex, depending on the deformation characteristics over the course of the treatment [[7], [17]]. For the GS simulation, DDM- and EMT-based accumulated doses did not show any significant differences. Thus, for the institutional comparison DDM was employed, as it is currently the more commonly used approach.

The study is subject to limitations; only one of the institutions used EMT for dose warping and one used DIR without contour guidance. This makes the comparison of algorithms challenging but also reflects the present clinical situation without clear recommendations for DDA usage. Furthermore, some of the employed software solutions lack regulatory approval, thereby restricting their clinical use.

In conclusion, the compared algorithms for DIR and DDA yielded a generally high level of agreement. Nevertheless, absolute differences of potentially clinically relevant magnitude were observed depending on the clinical cases and algorithms. Before using DDA for online adaptive MRgRT in clinical practice, further studies are needed to provide recommendations and guidelines. In addition, robust methods for uncertainty quantification should be further investigated to determine areas of variation in the future.

## Declaration of Competing Interest

MM and DT report institutional collaborations including financial and non-financial support by Elekta AB, Philips, TheraPanacea, Dr. Sennewald, Brainlab and PTW Freiburg.

MM acknowledges funding through the German Research Council (DFG), grants no. MU 4603/1-1 (PAK997/1) and ZI 736/2-1.

HZ is partly supported by the grant R01-EB028324 from National Institute of Biomedical Imaging andBioengineering, NIH.

All other authors do not declare financial interests/personal relationships.
