# Supplementary material for: A multi-institutional comparison of retrospective deformable dose accumulation for online adaptive magnetic resonance-guided radiotherapy
Source: Phys Imaging Radiat Oncol. 2024 May 17;30:100588. doi: 10.1016/j.phro.2024.100588 (PMC11176923; doi:10.1016/j.phro.2024.100588)
Supplement: Supplementary data 1 [file mmc1.docx]

Supplementary material

**Supplementary A: Material and Methods**


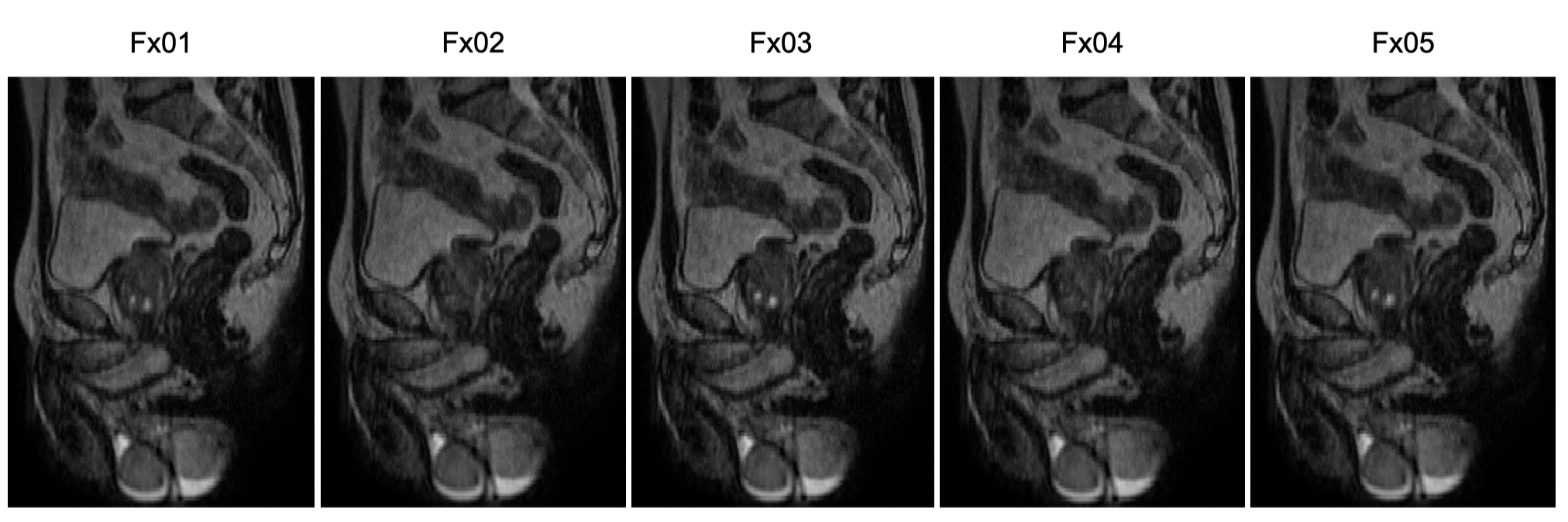


Supplementary Figure A.1: Illustration of the five deformed images, which resulted from the FEM experiment, in a representative sagittal slice.

Supplementary Table A.1: Specific MR images, dose files, and SBRT treatment information for the gold standard (GS), and the clinical cases; (I) prostate 1, (II) prostate 2, (III) cervix, (IV) liver, and (V) lymph node. Abbreviations; TR: repetition time TE: echo time. * MR image acquisition type: 4D, a MidP image was created for planning, and a motion-averaged 3D MR image from the 4D MR image was used for DIR [1].

| Specific | MR Image | | | | | | | | | | | | Dose | | | | SBRT  Treatment | |
| --- | --- | --- | --- | --- | --- | --- | --- | --- | --- | --- | --- | --- | --- | --- | --- | --- | --- | --- |
| information  Cases | MR Image Type for DIR | TR  [ms] | TE  [ms] | Pixel Bandwith  [Hz/Px] | Flip Angle  [°] | Acquisition Matrix | Number of  Frames | Rows | Columns | Pixel  Spacing  [mm] | Slice  Thickness  [mm] | Spacing  between Slices  [mm] | Number of  Frames | Rows | Columns | Pixel  Spacing  [mm] | Fractions | Dose  [Gy] |
| Gold Standard | 3D | 1535 | 277 | 740 | 90 | 268 x 268 | 150 | 480 | 480 | 0.83 x 0.83 | 2 | 2 | 100 | 150 | 217 | 3 x 3 | 5 | 7.25 |
| (I) Prostate 1 | 3D | 1535 | 277 | 740 | 90 | 268 x 268 | 150 | 480 | 480 | 0.83 x 0.83 | 2 | 2 | 100 | 150 | 217 | 3 x 3 | 5 | 7.25 |
| (II) Prostate 2 | 3D | 1535 | 277 | 740 | 90 | 268 x 268 | 150 | 480 | 480 | 0.83 x 0.83 | 2 | 2 | 100 | 150 | 217 | 3 x 3 | 5 | 7.25 |
| (III) Cervix | 3D | 1400 | 150 | 694 | 90 | 497 x 497 | 125 | 672 | 672 | 0.8 x 0.8 | 2 | 2 | 85 | 188 | 217 | 3 x 3 | 5 | 7 |
| (IV) Liver | 3D* | 4.3 | 2.071 | 775 | 50 | 256 x 256 | 84 | 256 | 256 | 1.64 x 1.64 | 2.38 | 2.38 | 67 | 190 | 217 | 3 x 3 | 5 | 7 |
| (V) Lymph node | 3D | 1535 | 277 | 740 | 90 | 268 x 268 | 300 | 480 | 480 | 0.83 x 0.83 | 2 | 1 | 150 | 251 | 325 | 2 x 2 | 5 | 6 |


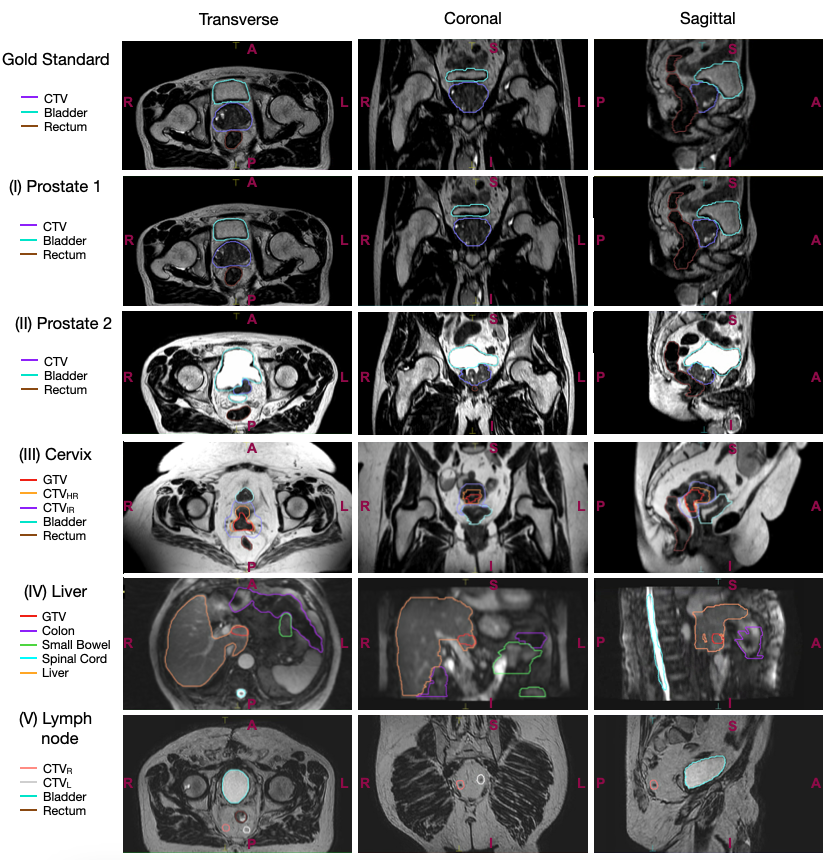


Supplementary Figure A.2: Illustration of the golds standard (GS), and the clinical cases; (I) prostate 1, (II) prostate 2, (III) cervix, (IV) liver, and (V) lymph node. The illustration shows one slice of transverse, coronal and sagittal view of the first fraction (Fx01) of each case. The different contours per case show the offline recontoured contours of Fx01. Note that for the GS, prostate 1 fraction 1 was used as base image. Abbreviations; CTV: clinical target volume, GTV: gross tumor volume, CTV_HR_: High risk clinical target volume, CTV_IR_: Intermediate risk clinical target volume, R: right, L: left, P: posterior, A: anterior, I: inferior and S: superior.

Supplementary Table A.2: Clinical dosimetric criteria (CDC) results, fraction-based and deformable dose accumulation (DDA) of the institutes for the gold standard (GS) and the clinical cases; (I) prostate 1, (II) prostate 2, (III) cervix, (IV) liver, and (V) lymph nodes. The respective CDC for the individual institutes DDA were calculated by ProKnow by the use of offline re-contoured structure set of fraction one (Fx01). Abbreviations; CTV: clinical target volume, GTV: gross tumor volume, CTV_HR_: High risk clinical target volume, CTV_IR_: Intermediate risk clinical target volume, CTV_L_: left clinical target volume, CTV_R_: right clinical target volume, Sum: summed dose.

| Case | Clinical dosimetric criteria (CDC) | | | | Fraction based | | | | | | Accumulated | | | | | | | |
| --- | --- | --- | --- | --- | --- | --- | --- | --- | --- | --- | --- | --- | --- | --- | --- | --- | --- | --- |
|  |  |  |  |  | Fx01 | Fx02 | Fx03 | Fx04 | Fx05 | Sum | A | B | C | D | E | F | Mean | Std |
| Gold Standard | CTV | V34.4Gy | ≥ 99.0 | [%] | 99.8 | 83.4 | 99.9 | 97.5 | 100.0 | 96.1 | 86.3 | 93.1 | 92.6 | 92.9 | 95.8 | 92.0 | 92.1 | 3.1 |
|  | Bladder | D5cm^3^ | < 37.0 | [Gy] | 36.9 | 37.8 | 34.4 | 35.6 | 35.6 | 36.1 | 34.6 | 34.0 | 34.2 | 34.2 | 33.0 | 33.8 | 34.0 | 0.5 |
|  |  | V28.0Gy | < 15.0 | [%] | 10.9 | 15.9 | 8.7 | 6.1 | 8.0 | 9.9 | 8.6 | 8.0 | 7.9 | 7.9 | 8.1 | 7.7 | 8.0 | 0.3 |
|  |  | V32.0Gy | < 20.0 | [%] | 7.5 | 12.0 | 4.9 | 3.9 | 5.1 | 6.7 | 4.7 | 4.1 | 4.1 | 4.1 | 3.2 | 3.9 | 4.0 | 0.5 |
|  | Rectum | D1cm^3^ | ≤  38.0 | [Gy] | 36.3 | 27.6 | 35.9 | 35.8 | 37.7 | 34.6 | 33.0 | 34.0 | 34.1 | 34.0 | 35.3 | 33.9 | 34.1 | 0.7 |
|  |  | V28.0Gy | ≤ 15.0 | [%] | 12.1 | 1.4 | 14.8 | 12.4 | 17.3 | 11.6 | 9.8 | 10.2 | 10.3 | 9.8 | 12.5 | 9.8 | 10.4 | 1.1 |
|  |  | V32.0Gy | ≤  20.0 | [%] | 8.5 | 0.3 | 10.6 | 8.4 | 13.0 | 8.2 | 3.5 | 4.8 | 4.9 | 4.5 | 7.6 | 4.2 | 4.9 | 1.4 |
| (I) Prostate1 | CTV | V34.4Gy | ≥ 99.0 | [%] | 100.0 | 99.7 | 100.0 | 100.0 | 100.0 | 99.9 | 100.0 | 100.0 | 100.0 | 99.9 | 100.0 | 100.0 | 100.0 | 0.0 |
|  | Bladder | D5cm^3^ | < 37.0 | [Gy] | 34.7 | 31.7 | 36.0 | 36.3 | 36.3 | 35.0 | 33.4 | 35.8 | 34.0 | 31.9 | 36.5 | 33.8 | 34.2 | 1.7 |
|  |  | V28.0Gy | < 15.0 | [%] | 7.6 | 2.8 | 10.5 | 5.5 | 12.6 | 7.8 | 7.6 | 11.3 | 8.1 | 5.9 | 16.1 | 7.5 | 9.4 | 3.7 |
|  |  | V32.0Gy | < 20.0 | [%] | 4.5 | 1.2 | 6.8 | 3.7 | 8.4 | 4.9 | 3.8 | 7.1 | 4.5 | 2.5 | 10.5 | 4.1 | 5.4 | 2.9 |
|  | Rectum | D1cm^3^ | ≤  38.0 | [Gy] | 37.3 | 37.6 | 36.6 | 37.7 | 37.2 | 37.3 | 36.4 | 35.8 | 36.5 | 36.7 | 36.5 | 36.3 | 36.4 | 0.3 |
|  |  | V28.0Gy | ≤ 15.0 | [%] | 15.5 | 15.7 | 12.9 | 16.0 | 10.9 | 14.2 | 14.0 | 14.0 | 14.6 | 14.5 | 14.7 | 13.9 | 14.3 | 0.4 |
|  |  | V32.0Gy | ≤  20.0 | [%] | 11.8 | 12.2 | 9.2 | 12.3 | 6.5 | 10.4 | 9.6 | 9.4 | 10.4 | 10.4 | 9.6 | 9.0 | 9.7 | 0.6 |
| (II) Prostate2 | CTV | V34.4Gy | ≥ 99.0 | [%] | 99.8 | 100.0 | 99.5 | 100.0 | 100.0 | 99.9 | 100.0 | 100.0 | 100.0 | 100.0 | 97.1 | 100.0 | 99.5 | 1.2 |
|  | Bladder | D5cm^3^ | < 37.0 | [Gy] | 36.8 | 36.6 | 36.6 | 36.7 | 36.4 | 36.6 | 36.3 | 36.2 | 36.3 | 36.3 | 36.5 | 36.2 | 36.3 | 0.1 |
|  |  | V28.0Gy | < 15.0 | [%] | 30.4 | 31.9 | 18.4 | 23.0 | 20.5 | 24.8 | 22.8 | 22.4 | 22.7 | 25.6 | 30.0 | 22.7 | 24.4 | 3.0 |
|  |  | V32.0Gy | < 20.0 | [%] | 22.6 | 23.0 | 12.9 | 16.0 | 14.9 | 17.9 | 14.8 | 14.1 | 14.7 | 16.8 | 21.5 | 14.5 | 16.1 | 2.8 |
|  | Rectum | D1cm^3^ | ≤  38.0 | [Gy] | 33.6 | 35.3 | 36.1 | 36.8 | 36.0 | 35.6 | 34.7 | 34.8 | 34.0 | 35.0 | 32.8 | 34.9 | 34.4 | 0.8 |
|  |  | V28.0Gy | ≤ 15.0 | [%] | 6.6 | 9.8 | 11.5 | 16.7 | 9.1 | 10.7 | 9.0 | 8.4 | 8.1 | 10.7 | 5.5 | 9.5 | 8.5 | 1.7 |
|  |  | V32.0Gy | ≤  20.0 | [%] | 3.0 | 5.2 | 6.1 | 11.3 | 4.8 | 6.1 | 4.7 | 4.6 | 3.7 | 5.5 | 2.2 | 5.2 | 4.3 | 1.2 |
| (III) Cervix | GTV | D98% | > 35.0 | [Gy] | 35.8 | 35.5 | 34.4 | 35.6 | 34.4 | 35.1 | 34.9 | 35.2 | 34.8 | 34.7 | 33.8 | 34.6 | 34.7 | 0.5 |
|  | CTV_HR_ | D90% | > 33.0 | [Gy] | 33.6 | 33.7 | 33.2 | 33.4 | 28.2 | 32.4 | 33.0 | 33.0 | 32.9 | 33.0 | 32.9 | 33.0 | 33.0 | 0.1 |
|  | CTV_HR_ | D98% | > 25.0 | [Gy] | 32.0 | 32.0 | 31.6 | 31.8 | 25.7 | 30.6 | 31.0 | 30.9 | 31.0 | 30.9 | 31.1 | 31.2 | 31.0 | 0.1 |
|  | CT_IR_ | D98% | > 14.5 | [Gy] | 24.9 | 24.4 | 24.6 | 25.1 | 18.2 | 23.4 | 25.3 | 25.4 | 25.2 | 25.7 | 23.8 | 25.2 | 25.1 | 0.7 |
|  | Bladder | D2cm^3^ | < 27.5 | [Gy] | 26.7 | 27.8 | 27.0 | 27.3 | 26.0 | 27.0 | 26.9 | 26.9 | 26.9 | 26.4 | 27.0 | 26.7 | 26.8 | 0.2 |
|  | Rectum | D2cm^3^ | < 21.5 | [Gy] | 16.5 | 19.4 | 14.3 | 21.3 | 15.9 | 17.5 | 17.4 | 17.1 | 17.3 | 19.3 | 16.5 | 17.7 | 17.6 | 0.9 |
|  | Sigmoid | D2cm^3^ | < 21.5 | [Gy] | 21.4 | 20.7 | 22.0 | 20.9 | 22.6 | 21.5 | 21.1 | 22.1 | 20.9 | 23.5 | 21.7 | 21.1 | 21.7 | 1.0 |
| (IV) Liver | Colon | Dmax | < 32.0 | [Gy] | 21.7 | 19.5 | 21.0 | 18.3 | 20.1 | 19.7 | 22.6 | 23.5 | 22.9 | 19.7 | 17.1 | 22.8 | 21.4 | 2.5 |
|  | Duodenum | Dmax | < 30.0 | [Gy] | 21.8 | 20.7 | 24.0 | 32.4 | 18.4 | 23.9 | 23.7 | 22.5 | 23.0 | 17.7 | 16.6 | 22.8 | 21.1 | 3.1 |
|  | Small bowel | Dmax | < 30.0 | [Gy] | 11.4 | 16.4 | 14.9 | 15.5 | 11.9 | 14.7 | 12.7 | 10.2 | 11.0 | 10.2 | 11.3 | 11.0 | 11.1 | 0.9 |
|  | Spinal cord | Dmax | < 22.5 | [Gy] | 5.2 | 6.4 | 5.3 | 5.7 | 6.0 | 5.9 | 5.4 | 5.5 | 5.4 | 5.1 | 5.3 | 5.4 | 5.4 | 0.1 |
|  | Stomach | Dmax | < 30.0 | [Gy] | 31.8 | 28.2 | 33.1 | 32.3 | 30.6 | 31.1 | 28.9 | 29.8 | 29.3 | 26.3 | 26.6 | 29.8 | 28.5 | 1.6 |
| (V) Lymphnode | CTV_L_ | D0.5cm^3^ | < 42.0 | [Gy] | 41.5 | 41.8 | 41.2 | 41.9 | 41.8 | 41.7 | 40.8 | 39.3 | 40.9 | 38.2 | 40.1 | 41.5 | 40.1 | 1.2 |
|  |  | D98% | > 30.0 | [Gy] | 31.8 | 32.8 | 33.2 | 30.5 | 31.8 | 32.1 | 37.4 | 33.6 | 39.6 | 33.1 | 37.5 | 38.6 | 36.6 | 2.7 |
|  | CTV_R_ | D0.5cm^3^ | < 42.0 | [Gy] | 41.3 | 41.1 | 41.5 | 41.9 | 42.5 | 41.8 | 41.0 | 40.6 | 40.6 | 37.4 | 41.1 | 41.7 | 40.4 | 1.5 |
|  |  | D98% | > 30.0 | [Gy] | 30.5 | 31.8 | 31.8 | 32.8 | 33.2 | 32.4 | 39.4 | 37.7 | 39.5 | 31.9 | 38.9 | 40.0 | 37.9 | 3.0 |
|  | Bladder | D0.5cm^3^ | < 32.0 | [Gy] | 8.9 | 10.7 | 12.8 | 8.0 | 7.6 | 9.8 | 9.0 | 9.0 | 9.0 | 9.1 | 9.3 | 9.0 | 9.1 | 0.1 |
|  | Rectum | D0.5cm^3^ | < 32.0 | [Gy] | 16.6 | 11.3 | 13.8 | 16.2 | 16.3 | 14.4 | 14.2 | 14.8 | 14.4 | 14.1 | 18.7 | 14.8 | 15.2 | 1.8 |
|  | Sigmoid | D0.5cm^3^ | < 32.0 | [Gy] | 0.7 | 0.7 | 1.0 | 5.6 | 2.0 | 2.3 | 1.6 | 1.4 | 1.8 | 1.2 | 1.5 | 1.5 | 1.5 | 0.2 |

Supplementary Table A.3: Overview of the software used by the institutes (A-F) for deformable image registration (DIR) and dose accumulation. DVF: deformation vector field, DDM: direct dose mapping, EMT: Energy mass transfer.

| Institute | A | B | C | D | E | F |
| --- | --- | --- | --- | --- | --- | --- |
| Software (Version) | Monaco Research ADMIRE  (3.44.2)  for DIR and  Slicer 3D  (4.11)  for dose mapping/  accumulation | MIM  (6.8.5) | Raystation  (8B)  ANACONDA | MIM  (7.0.6) | In-house developed dual-force demons deformable registration | RRTracker  (v4.0) |
| DIR  algorithm | Hybrid  intensity /  structure based | Mixed / hybrid  DIR | Hybrid  intensity /  structure based | Contour based Deformable | Intensity  based | Normalized gradient fields / structure based |
| DIR  Contour guidance | Yes | Yes | Yes | Yes | No | Yes |
| DIR  transformation | DVF | DVF | DVF | DVF | DVF | DVF |
| Contour propagation  transformation | DVF | DVF | DVF | DVF | DVF | DVF |
| Dose  transformation | DVF | DVF | DVF | DVF | DVF | DVF |
| Dose  method | DDM | DDM | DDM | DDM | DDM | EMT |

Deformable image registration / Contour propagation / Dose accumulation software:

Institute A used Monaco ADMIRE Research (Elekta, Stockholm, Sweden) for deformable image registration and contour propagation. The software used an Efficient Inverse-Consistent Diffeomorphic Image Registration Method [2]. Case-specifically selected contours at the first fraction were used for guidance. The offline re-contoured contours of Fx02-Fx05 were propagated to Fx01 during DIR by using the corresponding DVF. For the dose mapping and accumulation 3D slicer [3] an open software tool was used. The doses of each fraction were mapped according to the DVF to the first fraction of the treatment. The accumulated dose was calculated by weighting the deformed doses by 20%.

Institute B: Hybrid deformable image registration involves registrations using contours as well as image intensity. For contour-based deformable registration, MIM iteratively minimizes the signed distance differences between the two images, as measured from the surface of contour pairs. For intensity-based registration, intensity differences between the two images are minimized using a constrained, free-form deformable registration based on intensity. For the hybrid mode, the influence of the contour surface matching decreases with increasing distance from the contour surface. As part of DIR workflow, the MRI of Fx01 was selected as reference to which all other images were deformably registered, using an appropriate set of contours as well as image intensity. The DVF generated for the DIR was used to deform the associated dose for each case. A dose accumulation was then performed by equally weighting each of the fractional doses included in the registration to achieve the prescribed dose. No changes in the DIR protocols were made during the study. However, two different versions of MIM were used; with the last dataset being processed with version 7.2.7.

Institute C: We employed a hybrid intensity and structure-based DIR approach called ANAtomically CONstrained Deformation Algorithm (ANACONDA) integrated in the RayStation 8B treatment planning system. This algorithm computes a deformation vector field by optimizing both image similarity and alignment of controlling ROI structures segmented a priori on the registered images. The optimization includes regularization terms to control smoothness and invertibility as well as penalize generation of large ROI deviations. The optimization employs a nonlinear limited memory solver applied sequentially over 10-, 5- and 2.5-mm resolutions, with result of each resolution used as the starting point for the next level. We employed default settings in RayStation to generate each DIR and used the deformation vector field to map the dose for each fraction back to the first fraction image. Accumulation was performed by weighting each fractional dose by 1/total number of fractions and then performing a summation of all fractional doses.

Institute D used the contour-only deformable registration for this study. The registration starts from an image intensity-based rigid registration and followed by a deformable registration that iteratively minimizes the signed distance between the surfaces of contour pairs in the two images. The default parameters in MIM were used for both the clinical and gold standard cases without any modification. However, individual contours used in each registration may vary. Details are specified in the corresponding figures.

Institute E: The daily MR images (Fx02, …, Fx05) were deformably registered to the first fraction MR image (Fx01) using a dual-force demons deformable registration [4]. The “Demons” algorithm introduced demons force from thermodynamics into image domain for deformable registration [5]. The demons force is driven by image gradients and image intensity differences and is updated iteratively and successively over entire image domain followed by a Gaussian-type kernel to smooth the force vectors and to propagate the forces to areas with low image gradients [6]. The dual-force demons algorithm was first developed for CT-CT deformable registration and was later validated for MR-MR deformable registration [7]. For this application, the algorithm parameters were set to allow only small deformation. The resulting deformation vector fields were used to deform the daily dose to first fraction and the deformed dose was then scaled to a fraction dose for accumulation [8–10].

Institute F: The employed dose accumulation solution relied on a variational registration algorithm, estimating displacements as the minimizer of a cost function composed of three terms: an image similarity term, a contour matching term and a regularization term. In summary, the image similarity term uses normalized gradient fields [11] in order to match similar contrast patterns between the registered images, whereas the contour matching term penalizes misalignments between any particular contour pairs defined by the user on the images. Finally, the regularization term constrains the estimated deformations to be spatially smooth, ensuring the well-posedness of the minimization problem. Further details regarding the method can be found here [12]. The deformations estimated by the DIR algorithm were subsequently used to warp each daily dose distribution onto the reference grid, via energy-per-mass transfer [13], with the warped doses being added-up in order to generate the accumulated dose.

Data analysis:

The DSC is defined by

$DSC(X,Y)=\frac{2|X\cap Y|}{|X|+|Y|}$, (1)

where $X, Y$ are the two volume sets to be compared. AAPM TG132 [14] indicates a DSC tolerance of 0.8–0.9, except for very large or very small volumes.

In addition, the SDSC, was calculated with a threshold value of 2 mm. The SDCS quantifies the similarity between two surfaces by measuring the overlap and mismatch between them.

Third, $HD$ was determined, which is defined as the greatest distance of a contour set $X$ to the closest point of the other set $Y$[15]

$HD(X,Y)=max(h(X,Y),h(Y,X))$, (2)

where h is defined by

$h\left( X,Y \right)=\max\min\left\| x-y \right\|$, (3)

$x\in X, y\in Y$.

Due to the outlier sensitivity of $HD$, the $HD95\%$ was calculated over the histogram of all distances given points on X and Y by excluding the 5% highest outliers [16].

**Supplementary B: Results**

Supplementary Table B.1: Statistical values of dose accumulation errors selected for the gold standard (GS) case for the participating institutes. Values are in [Gy] and are presented individually for CTV (prostate), bladder, and rectum. Abbreviations; CTV: clinical target volume, IQR: interquartile range.

| Institute | Volume of interest | Mean | Standard deviation | 5th  percentile | 95th  percentile | Median | IQR |
| --- | --- | --- | --- | --- | --- | --- | --- |
| A | CTV  (Prostate) | 0.09 | 0.24 | -0.24 | 0.50 | 0.05 | 0.16 |
| B |  | 0.30 | 0.46 | -0.18 | 1.31 | 0.10 | 0.45 |
| C |  | 0.30 | 0.45 | -0.18 | 1.22 | 0.10 | 0.49 |
| D |  | 0.28 | 0.42 | -0.18 | 1.20 | 0.10 | 0.44 |
| E |  | 0.39 | 0.85 | -0.52 | 2.22 | 0.16 | 0.65 |
| F |  | 0.27 | 0.40 | -0.18 | 1.08 | 0.11 | 0.44 |
| A | Bladder | 0.40 | 0.76 | -0.58 | 2.02 | 0.16 | 0.71 |
| B |  | 0.41 | 0.67 | -0.50 | 1.73 | 0.22 | 0.73 |
| C |  | 0.47 | 0.72 | -0.49 | 1.89 | 0.24 | 0.81 |
| D |  | 0.12 | 0.74 | -0.85 | 1.51 | 0.00 | 0.46 |
| E |  | 0.68 | 1.84 | -1.40 | 4.38 | 0.18 | 1.05 |
| F |  | 0.40 | 0.67 | -0.63 | 1.68 | 0.24 | 0.74 |
| A | Rectum | 0.21 | 0.43 | -0.33 | 1.02 | 0.02 | 0.47 |
| B |  | 0.37 | 0.51 | -0.20 | 1.44 | 0.12 | 0.62 |
| C |  | 0.32 | 0.53 | -0.26 | 1.45 | 0.05 | 0.59 |
| D |  | 0.23 | 0.44 | -0.26 | 1.20 | 0.00 | 0.40 |
| E |  | 2.92 | 2.29 | 0.03 | 7.31 | 2.84 | 3.86 |
| F |  | 0.25 | 0.50 | -0.40 | 1.22 | 0.11 | 0.49 |

Supplementary Table B.2: Gold standard (GS) contour propagation evaluation metrics, DSC (Dice similarity coefficient), SDSC (surface DSC) with 2 mm threshold, and HD95% (Hausdorff distance 95%) for the contours and deformed fraction. G (contour guidance; if checked the contour was used as guidance by the institute. DSC printed in red if < 0.8. Abbreviations; Femur_L_: left femur, Femur_R_: right femur_,_ CTV: clinical target volume, GTV: gross tumor volume.

|  | | | **A** | | | | **C** | | | | **D** | | | | **E** | | | | **F** | | | |
| --- | --- | --- | --- | --- | --- | --- | --- | --- | --- | --- | --- | --- | --- | --- | --- | --- | --- | --- | --- | --- | --- | --- |
|  |  |  | **DSC** | **SDSC** | **HD95% [mm]** | **G** | **DSC** | **SDSC** | **HD95% [mm]** | **G** | **DSC** | **SDSC** | **HD95% [mm]** | **G** | **DSC** | **SDSC** | **HD95% [mm]** | **G** | **DSC** | **SDSC** | **HD95% [mm]** | **G** |
| **Gold Standard** | **Bladder** | Fx02toFx01 | 0.99 | 0.42 | 5.27 | ✔️ | 0.99 | 0.47 | 5.07 | ✔️ | 0.97 | 0.43 | 4.49 | ✔️ | 0.98 | 0.38 | 5.36 |  | 0.98 | 0.65 | 3.74 | ✔️ |
|  |  | Fx03toFx01 | 0.99 | 0.42 | 5.27 |  | 0.99 | 0.49 | 5.16 |  | 0.98 | 0.42 | 4.25 |  | 0.98 | 0.40 | 2.64 |  | 0.99 | 0.69 | 4.01 |  |
|  |  | Fx04toFx01 | 0.99 | 0.42 | 5.24 |  | 0.99 | 0.49 | 5.03 |  | 0.97 | 0.41 | 4.53 |  | 0.96 | 0.33 | 5.41 |  | 0.99 | 0.61 | 4.18 |  |
|  |  | Fx05toFX01 | 0.99 | 0.42 | 5.26 |  | 0.99 | 0.49 | 5.24 |  | 0.97 | 0.41 | 4.45 |  | 0.98 | 0.38 | 2.58 |  | 0.99 | 0.64 | 4.00 |  |
|  | **Rectum** | Fx02toFx01 | 0.98 | 0.56 | 3.40 | ✔️ | 0.98 | 0.63 | 3.38 | ✔️ | 0.97 | 0.58 | 3.13 | ✔️ | 0.97 | 0.54 | 3.49 |  | 0.95 | 0.66 | 2.75 | ✔️ |
|  |  | Fx03toFx01 | 0.99 | 0.57 | 3.39 |  | 0.99 | 0.66 | 3.37 |  | 0.95 | 0.55 | 3.27 |  | 0.98 | 0.56 | 3.43 |  | 0.94 | 0.70 | 3.05 |  |
|  |  | Fx04toFx01 | 0.98 | 0.56 | 3.41 |  | 0.98 | 0.64 | 3.37 |  | 0.95 | 0.55 | 3.36 |  | 0.94 | 0.51 | 3.69 |  | 0.94 | 0.63 | 2.75 |  |
|  |  | Fx05toFX01 | 0.99 | 0.56 | 3.39 |  | 0.99 | 0.66 | 3.31 |  | 0.96 | 0.57 | 3.16 |  | 0.98 | 0.56 | 3.50 |  | 0.93 | 0.62 | 3.20 |  |
|  | **Sphincter** | Fx02toFx01 | 0.97 | 0.55 | 3.62 |  | 0.98 | 0.66 | 3.40 |  | 0.97 | 0.59 | 3.22 | ✔️ | 0.96 | 0.54 | 3.59 |  | 0.91 | 0.63 | 3.23 |  |
|  |  | Fx03toFx01 | 0.99 | 0.57 | 3.64 |  | 0.98 | 0.67 | 3.56 |  | 0.92 | 0.53 | 3.95 |  | 0.98 | 0.57 | 1.26 |  | 0.91 | 0.64 | 3.33 |  |
|  |  | Fx04toFx01 | 0.97 | 0.56 | 3.66 |  | 0.98 | 0.66 | 3.36 |  | 0.95 | 0.56 | 3.23 |  | 0.92 | 0.50 | 3.80 |  | 0.90 | 0.66 | 4.10 |  |
|  |  | Fx05toFX01 | 0.99 | 0.58 | 3.63 |  | 0.98 | 0.68 | 3.32 |  | 0.97 | 0.58 | 3.30 |  | 0.97 | 0.57 | 3.56 |  | 0.89 | 0.58 | 3.29 |  |
|  | **Femur_L_** | Fx02toFx01 | 1.00 | 0.50 | 4.24 | ✔️ | 1.00 | 0.62 | 4.12 |  | 0.99 | 0.52 | 3.98 |  | 0.98 | 0.48 | 4.31 |  | 0.97 | 0.70 | 2.73 |  |
|  |  | Fx03toFx01 | 1.00 | 0.49 | 4.24 |  | 1.00 | 0.62 | 4.15 |  | 0.89 | 0.20 | 5.31 |  | 0.99 | 0.49 | 4.32 |  | 0.98 | 0.83 | 3.20 |  |
|  |  | Fx04toFx01 | 1.00 | 0.50 | 4.23 |  | 1.00 | 0.62 | 4.13 |  | 0.87 | 0.18 | 7.24 |  | 0.95 | 0.41 | 4.75 |  | 0.95 | 0.58 | 3.29 |  |
|  |  | Fx05toFX01 | 1.00 | 0.50 | 4.24 |  | 1.00 | 0.62 | 4.13 |  | 0.88 | 0.19 | 5.09 |  | 0.99 | 0.48 | 4.31 |  | 0.97 | 0.70 | 2.75 |  |
|  | **Femur_R_** | Fx02toFx01 | 1.00 | 0.54 | 4.03 | ✔️ | 1.00 | 0.64 | 3.90 |  | 0.99 | 0.54 | 4.00 |  | 0.98 | 0.51 | 4.10 |  | 0.95 | 0.57 | 3.47 |  |
|  |  | Fx03toFx01 | 1.00 | 0.53 | 4.04 |  | 1.00 | 0.64 | 3.86 |  | 0.89 | 0.27 | 4.39 |  | 0.99 | 0.53 | 1.03 |  | 0.97 | 0.72 | 3.09 |  |
|  |  | Fx04toFx01 | 1.00 | 0.54 | 4.02 |  | 1.00 | 0.65 | 3.86 |  | 0.88 | 0.20 | 5.87 |  | 0.95 | 0.47 | 4.25 |  | 0.98 | 0.77 | 2.72 |  |
|  |  | Fx05toFX01 | 1.00 | 0.54 | 4.03 |  | 1.00 | 0.65 | 3.88 |  | 0.91 | 0.30 | 4.57 |  | 0.98 | 0.52 | 4.11 |  | 0.95 | 0.57 | 3.28 |  |
|  | **CTV** | Fx02toFx01 | 0.97 | 0.45 | 4.35 |  | 0.99 | 0.57 | 4.07 | ✔️ | 0.95 | 0.44 | 3.72 | ✔️ | 0.97 | 0.44 | 4.35 |  | 0.97 | 0.65 | 3.72 | ✔️ |
|  |  | Fx03toFx01 | 0.99 | 0.47 | 4.31 |  | 0.99 | 0.57 | 4.09 |  | 0.97 | 0.48 | 3.76 |  | 0.98 | 0.49 | 4.35 |  | 0.98 | 0.72 | 3.28 |  |
|  |  | Fx04toFx01 | 0.97 | 0.46 | 4.30 |  | 0.99 | 0.57 | 3.97 |  | 0.94 | 0.38 | 4.15 |  | 0.96 | 0.41 | 4.36 |  | 0.96 | 0.64 | 3.62 |  |
|  |  | Fx05toFX01 | 0.97 | 0.45 | 4.34 |  | 0.99 | 0.57 | 4.08 |  | 0.96 | 0.44 | 3.97 |  | 0.97 | 0.44 | 4.61 |  | 0.96 | 0.61 | 3.46 |  |
|  | **GTV** | Fx02toFx01 | 0.92 | 0.69 | 2.87 |  | 0.93 | 0.74 | 2.74 |  | 0.90 | 0.68 | 2.88 |  | 0.91 | 0.67 | 2.84 |  | 0.89 | 0.77 | 2.98 | ✔️ |
|  |  | Fx03toFx01 | 0.93 | 0.69 | 2.97 |  | 0.95 | 0.77 | 2.72 |  | 0.91 | 0.70 | 2.78 |  | 0.95 | 0.70 | 2.82 |  | 0.92 | 0.84 | 2.72 |  |
|  |  | Fx04toFx01 | 0.92 | 0.68 | 2.90 |  | 0.94 | 0.77 | 2.94 |  | 0.90 | 0.68 | 2.77 |  | 0.88 | 0.62 | 3.24 |  | 0.90 | 0.83 | 2.76 |  |
|  |  | Fx05toFX01 | 0.92 | 0.68 | 2.88 |  | 0.94 | 0.73 | 2.67 |  | 0.91 | 0.68 | 2.80 |  | 0.91 | 0.66 | 2.93 |  | 0.83 | 0.64 | 4.02 |  |

Supplementary Table B.3: (I) Prostate 1 contour propagation evaluation metrics, DSC (Dice similarity coefficient), SDSC (surface DSC) with 2 mm threshold, and HD95% (Hausdorff distance 95%) for the contours and deformed fraction. G (contour guidance; if checked the contour was used as guidance by the institute. DSC printed in red if < 0.8. Abbreviations; Femur_L_: left femur, Femur_R_: right femur_,_ CTV: clinical target volume, GTV: gross tumor volume.

|  | | | **A** | | | | **C** | | | | **D** | | | | **E** | | | | **F** | | | |
| --- | --- | --- | --- | --- | --- | --- | --- | --- | --- | --- | --- | --- | --- | --- | --- | --- | --- | --- | --- | --- | --- | --- |
|  |  |  | **DSC** | **SDSC** | **HD95% [mm]** | **G** | **DSC** | **SDSC** | **HD95% [mm]** | **G** | **DSC** | **SDSC** | **HD95% [mm]** | **G** | **DSC** | **SDSC** | **HD95% [mm]** | **G** | **DSC** | **SDSC** | **HD95% [mm]** | **G** |
| **(I) Prostate 01** | **Bladder** | Fx02toFx01 | 0.99 | 0.74 | 3.10 | ✔️ | 0.98 | 0.73 | 3.38 | ✔️ | 0.91 | 0.54 | 4.31 | ✔️ | 0.70 | 0.23 | 17.50 |  | 0.98 | 0.58 | 5.81 | ✔️ |
|  |  | Fx03toFx01 | 0.99 | 0.75 | 3.05 |  | 0.99 | 0.79 | 3.29 |  | 0.97 | 0.64 | 3.53 |  | 0.96 | 0.55 | 4.12 |  | 0.99 | 0.65 | 4.31 |  |
|  |  | Fx04toFx01 | 0.99 | 0.74 | 3.06 |  | 0.98 | 0.73 | 3.26 |  | 0.94 | 0.51 | 4.19 |  | 0.85 | 0.25 | 8.02 |  | 0.98 | 0.67 | 4.13 |  |
|  |  | Fx05toFx01 | 0.99 | 0.75 | 3.11 |  | 0.99 | 0.80 | 3.22 |  | 0.97 | 0.64 | 3.73 |  | 0.97 | 0.58 | 2.59 |  | 0.99 | 0.63 | 4.56 |  |
|  | **Rectum** | Fx02toFx01 | 0.97 | 0.84 | 1.45 | ✔️ | 0.95 | 0.83 | 2.81 | ✔️ | 0.86 | 0.54 | 5.50 | ✔️ | 0.83 | 0.46 | 6.85 |  | 0.81 | 0.35 | 8.99 | ✔️ |
|  |  | Fx03toFx01 | 0.98 | 0.87 | 2.33 |  | 0.96 | 0.86 | 3.08 |  | 0.93 | 0.73 | 2.91 |  | 0.91 | 0.70 | 4.27 |  | 0.92 | 0.60 | 3.66 |  |
|  |  | Fx04toFx01 | 0.96 | 0.84 | 2.33 |  | 0.88 | 0.69 | 4.83 |  | 0.88 | 0.61 | 6.58 |  | 0.86 | 0.58 | 14.17 |  | 0.86 | 0.53 | 15.15 |  |
|  |  | Fx05toFx01 | 0.96 | 0.84 | 2.71 |  | 0.93 | 0.76 | 3.09 |  | 0.89 | 0.63 | 4.06 |  | 0.86 | 0.56 | 17.34 |  | 0.88 | 0.51 | 14.94 |  |
|  | **Sphincter** | Fx02toFx01 | 0.91 | 0.66 | 2.28 |  | 0.90 | 0.76 | 2.42 |  | 0.92 | 0.68 | 2.94 | ✔️ | 0.84 | 0.54 | 3.13 |  | 0.82 | 0.49 | 4.80 |  |
|  |  | Fx03toFx01 | 0.96 | 0.75 | 1.73 |  | 0.97 | 0.81 | 2.89 |  | 0.95 | 0.77 | 2.72 |  | 0.87 | 0.60 | 2.79 |  | 0.89 | 0.54 | 3.55 |  |
|  |  | Fx04toFx01 | 0.90 | 0.61 | 2.87 |  | 0.89 | 0.61 | 2.81 |  | 0.84 | 0.48 | 3.21 |  | 0.86 | 0.50 | 3.59 |  | 0.86 | 0.40 | 5.10 |  |
|  |  | Fx05toFx01 | 0.88 | 0.67 | 2.71 |  | 0.92 | 0.76 | 2.20 |  | 0.92 | 0.71 | 3.31 |  | 0.81 | 0.39 | 4.07 |  | 0.83 | 0.46 | 4.39 |  |
|  | **Femur_L_** | Fx02toFx01 | 1.00 | 0.90 | 2.18 | ✔️ | 0.98 | 0.93 | 2.24 |  | 0.77 | 0.26 | 10.68 |  | 0.97 | 0.89 | 1.17 |  | 0.95 | 0.61 | 4.14 |  |
|  |  | Fx03toFx01 | 0.99 | 0.90 | 2.17 |  | 0.98 | 0.93 | 2.25 |  | 0.91 | 0.63 | 4.67 |  | 0.97 | 0.88 | 2.30 |  | 0.95 | 0.57 | 4.26 |  |
|  |  | Fx04toFx01 | 1.00 | 0.90 | 2.18 |  | 0.99 | 0.93 | 2.18 |  | 0.83 | 0.36 | 10.37 |  | 0.97 | 0.89 | 1.32 |  | 0.95 | 0.62 | 4.15 |  |
|  |  | Fx05toFx01 | 0.99 | 0.90 | 2.18 |  | 0.96 | 0.89 | 2.35 |  | 0.89 | 0.51 | 4.67 |  | 0.95 | 0.81 | 2.62 |  | 0.94 | 0.50 | 4.13 |  |
|  | **Femur_R_** | Fx02toFx01 | 0.99 | 0.92 | 2.10 | ✔️ | 0.99 | 0.95 | 2.10 |  | 0.45 | 0.12 | 24.20 |  | 0.98 | 0.91 | 2.16 |  | 0.97 | 0.64 | 4.25 |  |
|  |  | Fx03toFx01 | 0.99 | 0.92 | 2.10 |  | 0.98 | 0.94 | 2.13 |  | 0.91 | 0.56 | 3.71 |  | 0.97 | 0.90 | 2.24 |  | 0.96 | 0.62 | 4.12 |  |
|  |  | Fx04toFx01 | 0.99 | 0.92 | 2.10 |  | 0.98 | 0.94 | 1.91 |  | 0.84 | 0.40 | 6.85 |  | 0.98 | 0.90 | 2.23 |  | 0.96 | 0.63 | 4.11 |  |
|  |  | Fx05toFx01 | 0.99 | 0.92 | 2.11 |  | 0.96 | 0.93 | 2.22 |  | 0.80 | 0.34 | 9.72 |  | 0.95 | 0.88 | 2.06 |  | 0.96 | 0.62 | 4.08 |  |
|  | **CTV** | Fx02toFx01 | 0.93 | 0.54 | 4.11 |  | 0.98 | 0.80 | 2.89 | ✔️ | 0.94 | 0.55 | 3.74 | ✔️ | 0.93 | 0.48 | 4.25 |  | 0.96 | 0.58 | 3.82 | ✔️ |
|  |  | Fx03toFx01 | 0.93 | 0.53 | 3.65 |  | 0.98 | 0.83 | 2.70 |  | 0.95 | 0.64 | 3.42 |  | 0.92 | 0.46 | 3.59 |  | 0.96 | 0.60 | 3.89 |  |
|  |  | Fx04toFx01 | 0.95 | 0.65 | 3.34 |  | 0.98 | 0.78 | 2.93 |  | 0.92 | 0.51 | 4.36 |  | 0.94 | 0.57 | 3.75 |  | 0.96 | 0.57 | 3.70 |  |
|  |  | Fx05toFx01 | 0.93 | 0.49 | 4.07 |  | 0.98 | 0.81 | 2.78 |  | 0.95 | 0.65 | 3.23 |  | 0.92 | 0.45 | 4.30 |  | 0.96 | 0.58 | 4.08 |  |
|  | **GTV** | Fx02toFx01 | 0.65 | 0.45 | 4.21 |  | 0.82 | 0.77 | 3.48 |  | 0.70 | 0.53 | 5.22 |  | 0.67 | 0.47 | 4.26 |  | 0.66 | 0.37 | 5.29 | ✔️ |
|  |  | Fx03toFx01 | 0.76 | 0.64 | 3.14 |  | 0.81 | 0.76 | 2.75 |  | 0.84 | 0.73 | 3.02 |  | 0.75 | 0.60 | 3.58 |  | 0.74 | 0.52 | 4.12 |  |
|  |  | Fx04toFx01 | 0.92 | 0.94 | 2.10 |  | 0.91 | 0.93 | 2.24 |  | 0.92 | 0.94 | 2.04 |  | 0.93 | 0.95 | 2.06 |  | 0.91 | 0.75 | 2.75 |  |
|  |  | Fx05toFx01 | 0.75 | 0.57 | 3.64 |  | 0.82 | 0.72 | 2.77 |  | 0.78 | 0.57 | 4.28 |  | 0.76 | 0.55 | 3.72 |  | 0.74 | 0.45 | 4.26 |  |

Supplementary Table B.4: (II) Prostate 2 contour propagation evaluation metrics, DSC (Dice similarity coefficient), SDSC (surface DSC) with 2 mm threshold, and HD95% (Hausdorff distance 95%) for the contours and deformed fraction. G (contour guidance; if checked the contour was used as guidance by the institute. DSC printed in red if < 0.8. Abbreviations; Femur_L_: left femur, Femur_R_: right femur_,_ CTV: clinical target volume, GTV: gross tumor volume.

|  | | | **A** | | | | **C** | | | | **D** | | | | **E** | | | | **F** | | | |
| --- | --- | --- | --- | --- | --- | --- | --- | --- | --- | --- | --- | --- | --- | --- | --- | --- | --- | --- | --- | --- | --- | --- |
|  |  |  | **DSC** | **SDSC** | **HD95% [mm]** | **G** | **DSC** | **SDSC** | **HD95% [mm]** | **G** | **DSC** | **SDSC** | **HD95% [mm]** | **G** | **DSC** | **SDSC** | **HD95% [mm]** | **G** | **DSC** | **SDSC** | **HD95% [mm]** | **G** |
| **(II) Prostate 02** | **Bladder** | Fx02toFx01 | 0.99 | 0.82 | 2.72 | ✔️ | 0.98 | 0.81 | 3.43 | ✔️ | 0.95 | 0.59 | 3.94 | ✔️ | 0.95 | 0.56 | 5.14 |  | 0.97 | 0.62 | 4.42 | ✔️ |
|  |  | Fx03toFx01 | 0.99 | 0.83 | 2.71 |  | 0.98 | 0.83 | 2.81 |  | 0.94 | 0.53 | 5.30 |  | 0.92 | 0.52 | 10.35 |  | 0.98 | 0.66 | 4.12 |  |
|  |  | Fx04toFx01 | 0.99 | 0.83 | 2.71 |  | 0.99 | 0.86 | 2.80 |  | 0.96 | 0.62 | 3.19 |  | 0.93 | 0.55 | 7.17 |  | 0.98 | 0.64 | 4.23 |  |
|  |  | Fx05toFx01 | 0.99 | 0.83 | 2.74 |  | 0.98 | 0.79 | 3.05 |  | 0.91 | 0.42 | 8.94 |  | 0.85 | 0.40 | 12.49 |  | 0.97 | 0.59 | 4.17 |  |
|  | **Rectum** | Fx02toFx01 | 0.98 | 0.86 | 2.38 | ✔️ | 0.84 | 0.73 | 15.17 | ✔️ | 0.87 | 0.51 | 4.62 | ✔️ | 0.81 | 0.52 | 12.79 |  | 0.87 | 0.43 | 5.54 | ✔️ |
|  |  | Fx03toFx01 | 0.97 | 0.85 | 1.81 |  | 0.97 | 0.87 | 2.47 |  | 0.82 | 0.47 | 7.05 |  | 0.83 | 0.55 | 11.25 |  | 0.81 | 0.40 | 10.50 |  |
|  |  | Fx04toFx01 | 0.98 | 0.87 | 0.97 |  | 0.97 | 0.89 | 2.45 |  | 0.78 | 0.44 | 7.32 |  | 0.93 | 0.71 | 2.62 |  | 0.89 | 0.51 | 3.37 |  |
|  |  | Fx05toFx01 | 0.98 | 0.86 | 1.08 |  | 0.96 | 0.86 | 2.56 |  | 0.78 | 0.39 | 9.65 |  | 0.74 | 0.38 | 15.12 |  | 0.84 | 0.40 | 9.81 |  |
|  | **Sphincter** | Fx02toFx01 | 0.81 | 0.51 | 3.36 |  | 0.78 | 0.55 | 3.71 |  | 0.82 | 0.59 | 2.87 | ✔️ | 0.79 | 0.40 | 3.90 |  | 0.85 | 0.51 | 4.27 |  |
|  |  | Fx03toFx01 | 0.79 | 0.59 | 3.35 |  | 0.78 | 0.68 | 3.51 |  | 0.83 | 0.62 | 3.67 |  | 0.79 | 0.46 | 3.70 |  | 0.84 | 0.55 | 3.83 |  |
|  |  | Fx04toFx01 | 0.73 | 0.49 | 5.10 |  | 0.73 | 0.49 | 5.43 |  | 0.77 | 0.52 | 5.12 |  | 0.70 | 0.34 | 5.44 |  | 0.64 | 0.35 | 5.77 |  |
|  |  | Fx05toFx01 | 0.75 | 0.51 | 5.60 |  | 0.76 | 0.55 | 5.87 |  | 0.72 | 0.51 | 4.05 |  | 0.68 | 0.27 | 6.55 |  | 0.72 | 0.37 | 5.19 |  |
|  | **Femur_L_** | Fx02toFx01 | 0.99 | 0.90 | 2.18 | ✔️ | 0.98 | 0.93 | 2.20 |  | 0.14 | 0.07 | 31.85 |  | 0.96 | 0.88 | 1.66 |  | 0.91 | 0.39 | 5.79 |  |
|  |  | Fx03toFx01 | 0.99 | 0.90 | 2.18 |  | 0.98 | 0.93 | 2.20 |  | 0.12 | 0.06 | 34.02 |  | 0.97 | 0.89 | 1.55 |  | 0.97 | 0.60 | 4.60 |  |
|  |  | Fx04toFx01 | 0.99 | 0.90 | 2.18 |  | 0.97 | 0.93 | 2.23 |  | 0.21 | 0.08 | 27.35 |  | 0.96 | 0.87 | 2.34 |  | 0.93 | 0.47 | 4.89 |  |
|  |  | Fx05toFx01 | 0.99 | 0.90 | 2.19 |  | 0.93 | 0.66 | 2.78 |  | 0.04 | 0.03 | 40.26 |  | 0.92 | 0.61 | 2.92 |  | 0.90 | 0.36 | 5.49 |  |
|  | **Femur_R_** | Fx02toFx01 | 0.99 | 0.90 | 2.14 | ✔️ | 0.90 | 0.50 | 4.11 |  | 0.59 | 0.16 | 18.36 |  | 0.89 | 0.49 | 4.20 |  | 0.83 | 0.20 | 5.65 |  |
|  |  | Fx03toFx01 | 0.99 | 0.91 | 2.14 |  | 0.96 | 0.90 | 2.32 |  | 0.37 | 0.09 | 25.70 |  | 0.96 | 0.86 | 2.40 |  | 0.96 | 0.55 | 4.68 |  |
|  |  | Fx04toFx01 | 0.99 | 0.91 | 2.14 |  | 0.97 | 0.94 | 2.20 |  | 0.65 | 0.14 | 12.68 |  | 0.97 | 0.88 | 2.27 |  | 0.96 | 0.57 | 4.91 |  |
|  |  | Fx05toFx01 | 0.99 | 0.91 | 2.13 |  | 0.95 | 0.89 | 2.32 |  | 0.34 | 0.08 | 24.02 |  | 0.95 | 0.85 | 2.43 |  | 0.94 | 0.46 | 4.74 |  |
|  | **CTV** | Fx02toFx01 | 0.93 | 0.56 | 2.76 |  | 0.98 | 0.85 | 2.61 | ✔️ | 0.89 | 0.31 | 4.58 | ✔️ | 0.92 | 0.52 | 3.25 |  | 0.95 | 0.55 | 4.33 | ✔️ |
|  |  | Fx03toFx01 | 0.92 | 0.51 | 3.51 |  | 0.98 | 0.85 | 2.74 |  | 0.93 | 0.56 | 3.63 |  | 0.90 | 0.49 | 4.04 |  | 0.95 | 0.55 | 3.46 |  |
|  |  | Fx04toFx01 | 0.92 | 0.51 | 3.18 |  | 0.98 | 0.85 | 2.65 |  | 0.92 | 0.55 | 3.81 |  | 0.90 | 0.45 | 3.82 |  | 0.94 | 0.53 | 3.96 |  |
|  |  | Fx05toFx01 | 0.91 | 0.51 | 4.10 |  | 0.98 | 0.83 | 2.70 |  | 0.92 | 0.55 | 3.88 |  | 0.89 | 0.44 | 3.93 |  | 0.94 | 0.54 | 4.27 |  |
|  | **GTV** | Fx02toFx01 | 0.43 | 0.49 | 3.76 |  | 0.49 | 0.42 | 4.79 |  | 0.60 | 0.55 | 4.03 |  | 0.23 | 0.61 | 4.82 |  | 0.19 | 0.31 | 5.21 | ✔️ |
|  |  | Fx03toFx01 | 0.43 | 0.46 | 4.06 |  | 0.40 | 0.30 | 6.07 |  | 0.16 | 0.25 | 6.85 |  | 0.26 | 0.52 | 5.25 |  | 0.23 | 0.28 | 4.88 |  |
|  |  | Fx04toFx01 | 0.19 | 0.53 | 6.80 |  | 0.16 | 0.49 | 8.06 |  | 0.14 | 0.43 | 8.17 |  | 0.12 | 0.38 | 7.57 |  | 0.06 | 0.45 | 10.12 |  |
|  |  | Fx05toFx01 | 0.73 | 0.76 | 3.38 |  | 0.35 | 0.35 | 7.00 |  | 0.01 | 0.14 | 10.21 |  | 0.23 | 0.50 | 4.41 |  | 0.27 | 0.30 | 5.27 |  |

Supplementary Table B.5: (III) Cervix contour propagation evaluation metrics, DSC (Dice similarity coefficient), SDSC (surface DSC) with 2 mm threshold, and HD95% (Hausdorff distance 95%) for the contours and deformed fraction. G (contour guidance; if checked the contour was used as guidance by the institute. DSC printed in red if < 0.8. Abbreviations; GTV: gross tumor volume, CTV_HR_: High risk clinical target volume, CTV_IR_: Intermediate risk clinical target volume.

|  | | | **A** | | | | **C** | | | | **D** | | | | **E** | | | | **F** | | | |
| --- | --- | --- | --- | --- | --- | --- | --- | --- | --- | --- | --- | --- | --- | --- | --- | --- | --- | --- | --- | --- | --- | --- |
|  |  |  | **DSC** | **SDSC** | **HD95% [mm]** | **G** | **DSC** | **SDSC** | **HD95% [mm]** | **G** | **DSC** | **SDSC** | **HD95% [mm]** | **G** | **DSC** | **SDSC** | **HD95% [mm]** | **G** | **DSC** | **SDSC** | **HD95% [mm]** | **G** |
| **(III)  Cervix** | **Bladder** | Fx02toFx01 | 0.97 | 0.80 | 1.32 | ✔️ | 0.97 | 0.76 | 2.01 | ✔️ | 0.86 | 0.23 | 5.87 | ✔️ | 0.93 | 0.71 | 2.93 |  | 0.97 | 0.62 | 3.14 | ✔️ |
|  |  | Fx03toFx01 | 0.97 | 0.70 | 1.98 |  | 0.97 | 0.70 | 2.10 |  | 0.82 | 0.62 | 5.63 |  | 0.93 | 0.67 | 2.59 |  | 0.97 | 0.51 | 3.02 |  |
|  |  | Fx04toFx01 | 0.97 | 0.75 | 1.37 |  | 0.97 | 0.79 | 2.04 |  | 0.87 | 0.84 | 4.14 |  | 0.94 | 0.73 | 2.77 |  | 0.97 | 0.56 | 3.08 |  |
|  |  | Fx05toFx01 | 0.97 | 0.70 | 1.84 |  | 0.97 | 0.68 | 1.94 |  | 0.77 | 0.25 | 6.17 |  | 0.92 | 0.67 | 2.70 |  | 0.96 | 0.27 | 2.09 |  |
|  | **Rectum** | Fx02toFx01 | 0.97 | 0.64 | 1.94 | ✔️ | 0.97 | 0.61 | 1.84 | ✔️ | 0.76 | 0.23 | 7.35 | ✔️ | 0.90 | 0.58 | 2.59 |  | 0.93 | 0.69 | 3.12 | ✔️ |
|  |  | Fx03toFx01 | 0.97 | 0.57 | 1.98 |  | 0.97 | 0.56 | 1.84 |  | 0.79 | 0.59 | 4.34 |  | 0.90 | 0.57 | 2.39 |  | 0.94 | 0.62 | 3.09 |  |
|  |  | Fx04toFx01 | 0.97 | 0.67 | 1.94 |  | 0.97 | 0.67 | 1.80 |  | 0.79 | 0.78 | 4.78 |  | 0.92 | 0.65 | 2.22 |  | 0.92 | 0.62 | 3.18 |  |
|  |  | Fx05toFx01 | 0.97 | 0.07 | 2.00 |  | 0.97 | 0.09 | 1.88 |  | 0.69 | 0.11 | 7.78 |  | 0.92 | 0.08 | 2.21 |  | 0.93 | 0.32 | 3.09 |  |
|  | **Sigmoid** | Fx02toFx01 | 0.95 | 0.40 | 1.87 | ✔️ | 0.95 | 0.38 | 1.83 | ✔️ | 0.59 | 0.20 | 11.49 | ✔️ | 0.81 | 0.30 | 6.72 |  | 0.80 | 0.34 | 6.27 |  |
|  |  | Fx03toFx01 | 0.96 | 0.43 | 1.09 |  | 0.96 | 0.46 | 1.52 |  | 0.60 | 0.47 | 9.30 |  | 0.82 | 0.39 | 4.09 |  | 0.82 | 0.39 | 4.28 |  |
|  |  | Fx04toFx01 | 0.97 | 0.54 | 1.50 |  | 0.96 | 0.54 | 1.45 |  | 0.74 | 0.58 | 4.57 |  | 0.86 | 0.50 | 2.47 |  | 0.85 | 0.44 | 3.08 |  |
|  |  | Fx05toFx01 | 0.96 | 0.16 | 1.62 |  | 0.96 | 0.19 | 1.47 |  | 0.58 | 0.12 | 6.91 |  | 0.84 | 0.16 | 4.13 |  | 0.82 | 0.15 | 3.86 |  |
|  | **GTV** | Fx02toFx01 | 0.90 | 0.97 | 2.68 |  | 0.88 | 0.97 | 2.92 |  | 0.66 | 0.49 | 6.90 | ✔️ | 0.88 | 0.84 | 2.86 |  | 0.92 | 0.65 | 3.51 |  |
|  |  | Fx03toFx01 | 0.86 | 0.97 | 4.60 |  | 0.85 | 0.97 | 5.07 |  | 0.82 | 0.41 | 3.28 |  | 0.87 | 0.85 | 4.39 |  | 0.88 | 0.66 | 4.11 |  |
|  |  | Fx04toFx01 | 0.89 | 0.97 | 2.58 |  | 0.89 | 0.97 | 2.46 |  | 0.92 | 0.55 | 2.34 |  | 0.87 | 0.85 | 2.76 |  | 0.89 | 0.64 | 3.40 |  |
|  |  | Fx05toFx01 | 0.85 | 0.97 | 3.20 |  | 0.84 | 0.98 | 3.71 |  | 0.66 | 0.31 | 6.50 |  | 0.84 | 0.83 | 3.80 |  | 0.70 | 0.64 | 6.42 |  |
|  | **CTV_HR_** | Fx02toFx01 | 0.89 | 0.97 | 3.38 |  | 0.88 | 0.98 | 3.54 |  | 0.76 | 0.51 | 6.03 | ✔️ | 0.89 | 0.85 | 3.76 |  | 0.95 | 0.68 | 3.18 | ✔️ |
|  |  | Fx03toFx01 | 0.85 | 0.97 | 5.24 |  | 0.85 | 0.98 | 4.99 |  | 0.85 | 0.55 | 3.78 |  | 0.86 | 0.85 | 4.62 |  | 0.94 | 0.69 | 2.66 |  |
|  |  | Fx04toFx01 | 0.88 | 0.97 | 2.40 |  | 0.89 | 0.98 | 2.48 |  | 0.92 | 0.50 | 3.20 |  | 0.88 | 0.91 | 3.10 |  | 0.93 | 0.62 | 3.40 |  |
|  |  | Fx05toFx01 | 0.55 | 0.96 | 11.28 |  | 0.56 | 0.98 | 11.32 |  | 0.53 | 0.40 | 12.66 |  | 0.56 | 0.89 | 11.20 |  | 0.81 | 0.69 | 7.56 |  |
|  | **CTV_IR_** | Fx02toFx01 | 0.92 | 0.96 | 5.05 |  | 0.90 | 0.98 | 4.46 |  | 0.86 | 0.37 | 6.52 | ✔️ | 0.90 | 0.71 | 5.91 |  | 0.92 | 0.45 | 4.65 |  |
|  |  | Fx03toFx01 | 0.91 | 0.98 | 5.77 |  | 0.91 | 0.98 | 6.10 |  | 0.91 | 0.37 | 4.22 |  | 0.90 | 0.76 | 5.77 |  | 0.91 | 0.49 | 5.16 |  |
|  |  | Fx04toFx01 | 0.94 | 0.98 | 2.55 |  | 0.94 | 0.98 | 2.72 |  | 0.95 | 0.48 | 3.72 |  | 0.94 | 0.83 | 2.66 |  | 0.93 | 0.50 | 4.24 |  |
|  |  | Fx05toFx01 | 0.72 | 0.98 | 11.10 |  | 0.72 | 0.98 | 11.15 |  | 0.70 | 0.31 | 12.55 |  | 0.73 | 0.80 | 10.58 |  | 0.72 | 0.47 | 11.10 |  |

Supplementary Table B.6: (IV) Liver contour propagation evaluation metrics, DSC (Dice similarity coefficient), SDSC (surface DSC) with 2 mm threshold, and HD95% (Hausdorff distance 95%) for the contours and deformed fraction. G (contour guidance; if checked the contour was used as guidance by the institute. DSC printed in red if < 0.8. Abbreviations; Kidney_L_: left kidney, Kidney_R_: right kidney, GTV: gross tumor volume.

|  | | | **A** | | | | **C** | | | | **D** | | | | **E** | | | | **F** | | | |
| --- | --- | --- | --- | --- | --- | --- | --- | --- | --- | --- | --- | --- | --- | --- | --- | --- | --- | --- | --- | --- | --- | --- |
|  |  |  | **DSC** | **SDSC** | **HD95% [mm]** | **G** | **DSC** | **SDSC** | **HD95% [mm]** | **G** | **DSC** | **SDSC** | **HD95% [mm]** | **G** | **DSC** | **SDSC** | **HD95% [mm]** | **G** | **DSC** | **SDSC** | **HD95% [mm]** | **G** |
| **(IV) Liver** | **Stomach** | Fx02toFx01 | 0.95 | 0.86 | 3.17 | ✔️ | 0.78 | 0.65 | 4.57 | ✔️ | 0.51 | 0.18 | 13.58 |  | 0.70 | 0.43 | 8.01 |  | 0.75 | 0.25 | 7.30 | ✔️ |
|  |  | Fx03toFx01 | 0.95 | 0.83 | 2.50 |  | 0.77 | 0.61 | 5.09 |  | 0.59 | 0.22 | 10.43 |  | 0.66 | 0.39 | 11.78 |  | 0.75 | 0.24 | 7.29 |  |
|  |  | Fx04toFx01 | 0.95 | 0.84 | 3.27 |  | 0.78 | 0.66 | 4.33 |  | 0.61 | 0.27 | 10.51 |  | 0.70 | 0.47 | 9.92 |  | 0.76 | 0.26 | 7.41 |  |
|  |  | Fx05toFx01 | 0.94 | 0.81 | 3.18 |  | 0.77 | 0.61 | 5.38 |  | 0.62 | 0.22 | 9.53 |  | 0.68 | 0.45 | 12.84 |  | 0.75 | 0.25 | 9.42 |  |
|  | **Spleen** | Fx02toFx01 | 0.96 | 0.89 | 2.74 |  | 0.82 | 0.66 | 3.14 |  | 0.70 | 0.26 | 7.42 |  | 0.80 | 0.57 | 5.16 |  | 0.82 | 0.23 | 5.80 |  |
|  |  | Fx03toFx01 | 0.93 | 0.76 | 3.81 |  | 0.79 | 0.56 | 4.83 |  | 0.68 | 0.24 | 8.79 |  | 0.78 | 0.59 | 5.27 |  | 0.78 | 0.17 | 6.96 |  |
|  |  | Fx04toFx01 | 0.96 | 0.88 | 2.68 |  | 0.81 | 0.64 | 3.75 |  | 0.75 | 0.28 | 5.23 |  | 0.81 | 0.68 | 4.90 |  | 0.82 | 0.22 | 6.25 |  |
|  |  | Fx05toFx01 | 0.95 | 0.87 | 2.74 |  | 0.81 | 0.61 | 4.39 |  | 0.70 | 0.35 | 8.56 |  | 0.76 | 0.61 | 16.31 |  | 0.82 | 0.24 | 6.04 |  |
|  | **Spinal cord** | Fx02toFx01 | 0.87 | 0.83 | 1.69 | ✔️ | 0.80 | 0.80 | 9.53 |  | 0.64 | 0.51 | 7.28 | ✔️ | 0.83 | 0.80 | 3.27 |  | 0.79 | 0.47 | 4.48 |  |
|  |  | Fx03toFx01 | 0.87 | 0.27 | 4.89 |  | 0.53 | 0.27 | 7.08 |  | 0.51 | 0.26 | 8.24 |  | 0.51 | 0.27 | 7.71 |  | 0.50 | 0.13 | 8.39 |  |
|  |  | Fx04toFx01 | 0.88 | 0.81 | 0.83 |  | 0.83 | 0.81 | 2.62 |  | 0.68 | 0.49 | 4.67 |  | 0.84 | 0.81 | 2.77 |  | 0.81 | 0.51 | 3.70 |  |
|  |  | Fx05toFx01 | 0.88 | 0.80 | 0.80 |  | 0.81 | 0.78 | 2.88 |  | 0.60 | 0.46 | 6.50 |  | 0.81 | 0.79 | 2.95 |  | 0.79 | 0.49 | 3.56 |  |
|  | **Small bowel** | Fx02toFx01 | 0.82 | 0.72 | 19.92 | ✔️ | 0.65 | 0.30 | 17.53 |  | 0.60 | 0.20 | 16.83 | ✔️ | 0.68 | 0.33 | 18.61 |  | 0.58 | 0.09 | 19.45 |  |
|  |  | Fx03toFx01 | 0.85 | 0.72 | 7.34 |  | 0.65 | 0.25 | 17.27 |  | 0.61 | 0.16 | 15.66 |  | 0.61 | 0.28 | 20.38 |  | 0.62 | 0.09 | 22.67 |  |
|  |  | Fx04toFx01 | 0.85 | 0.69 | 5.84 |  | 0.55 | 0.22 | 19.06 |  | 0.52 | 0.12 | 18.41 |  | 0.60 | 0.29 | 17.69 |  | 0.54 | 0.07 | 22.00 |  |
|  |  | Fx05toFx01 | 0.84 | 0.67 | 6.22 |  | 0.61 | 0.29 | 14.73 |  | 0.51 | 0.18 | 15.10 |  | 0.61 | 0.34 | 19.50 |  | 0.40 | 0.09 | 21.57 |  |
|  | **Pancreas** | Fx02toFx01 | 0.80 | 0.64 | 6.44 |  | 0.93 | 0.87 | 3.20 | ✔️ | 0.57 | 0.26 | 8.69 | ✔️ | 0.87 | 0.73 | 4.49 |  | 0.81 | 0.24 | 8.71 |  |
|  |  | Fx03toFx01 | 0.80 | 0.62 | 5.65 |  | 0.93 | 0.87 | 3.26 |  | 0.63 | 0.35 | 8.01 |  | 0.84 | 0.68 | 6.06 |  | 0.80 | 0.22 | 7.98 |  |
|  |  | Fx04toFx01 | 0.82 | 0.67 | 4.86 |  | 0.95 | 0.91 | 2.65 |  | 0.73 | 0.37 | 7.03 |  | 0.88 | 0.77 | 4.12 |  | 0.83 | 0.29 | 8.20 |  |
|  |  | Fx05toFx01 | 0.76 | 0.54 | 6.21 |  | 0.94 | 0.87 | 2.65 |  | 0.58 | 0.32 | 11.06 |  | 0.82 | 0.63 | 5.57 |  | 0.77 | 0.24 | 10.52 |  |
|  | **Liver** | Fx02toFx01 | 0.90 | 0.60 | 4.96 |  | 0.85 | 0.74 | 2.74 | ✔️ | 0.76 | 0.19 | 9.62 |  | 0.82 | 0.47 | 6.15 |  | 0.83 | 0.15 | 7.14 |  |
|  |  | Fx03toFx01 | 0.85 | 0.63 | 18.47 |  | 0.85 | 0.75 | 3.66 |  | 0.73 | 0.20 | 10.68 |  | 0.80 | 0.42 | 8.71 |  | 0.79 | 0.14 | 14.18 |  |
|  |  | Fx04toFx01 | 0.91 | 0.67 | 5.00 |  | 0.85 | 0.74 | 3.39 |  | 0.75 | 0.15 | 9.64 |  | 0.83 | 0.60 | 4.67 |  | 0.83 | 0.18 | 7.00 |  |
|  |  | Fx05toFx01 | 0.91 | 0.73 | 3.90 |  | 0.85 | 0.77 | 3.06 |  | 0.75 | 0.18 | 10.26 |  | 0.83 | 0.65 | 4.69 |  | 0.84 | 0.19 | 3.77 |  |
|  | **Kidney_R_** | Fx02toFx01 | 0.82 | 0.69 | 3.01 | ✔️ | 0.84 | 0.69 | 10.61 |  | 0.69 | 0.26 | 13.26 | ✔️ | 0.89 | 0.76 | 10.26 |  | 0.87 | 0.22 | 11.15 |  |
|  |  | Fx03toFx01 | 0.84 | 0.70 | 2.18 |  | 0.84 | 0.77 | 4.81 |  | 0.66 | 0.22 | 11.96 |  | 0.87 | 0.80 | 3.73 |  | 0.82 | 0.24 | 7.46 |  |
|  |  | Fx04toFx01 | 0.84 | 0.77 | 2.21 |  | 0.87 | 0.79 | 3.87 |  | 0.71 | 0.29 | 9.33 |  | 0.87 | 0.81 | 4.46 |  | 0.85 | 0.29 | 7.03 |  |
|  |  | Fx05toFx01 | 0.84 | 0.76 | 2.12 |  | 0.87 | 0.82 | 2.96 |  | 0.71 | 0.27 | 8.60 |  | 0.88 | 0.86 | 2.74 |  | 0.86 | 0.29 | 6.25 |  |
|  | **Kidney_L_** | Fx02toFx01 | 0.83 | 0.77 | 3.44 | ✔️ | 0.81 | 0.80 | 19.71 |  | 0.67 | 0.30 | 12.61 | ✔️ | 0.87 | 0.75 | 10.27 |  | 0.86 | 0.24 | 14.59 |  |
|  |  | Fx03toFx01 | 0.85 | 0.66 | 2.30 |  | 0.88 | 0.78 | 3.75 |  | 0.80 | 0.36 | 6.37 |  | 0.88 | 0.76 | 5.30 |  | 0.87 | 0.22 | 6.24 |  |
|  |  | Fx04toFx01 | 0.85 | 0.78 | 2.31 |  | 0.92 | 0.86 | 3.26 |  | 0.85 | 0.53 | 4.78 |  | 0.92 | 0.87 | 2.88 |  | 0.92 | 0.30 | 2.66 |  |
|  |  | Fx05toFx01 | 0.85 | 0.81 | 2.06 |  | 0.91 | 0.91 | 2.48 |  | 0.79 | 0.38 | 6.63 |  | 0.91 | 0.88 | 2.68 |  | 0.92 | 0.31 | 5.38 |  |
|  | **GTV** | Fx02toFx01 | 0.71 | 0.45 | 5.29 |  | 0.79 | 0.85 | 2.32 | ✔️ | 0.26 | 0.26 | 12.00 | ✔️ | 0.49 | 0.31 | 5.75 |  | 0.76 | 0.49 | 4.45 | ✔️ |
|  |  | Fx03toFx01 | 0.73 | 0.48 | 5.49 |  | 0.79 | 0.78 | 3.12 |  | 0.44 | 0.45 | 8.53 |  | 0.60 | 0.35 | 5.29 |  | 0.74 | 0.32 | 5.74 |  |
|  |  | Fx04toFx01 | 0.75 | 0.52 | 6.90 |  | 0.79 | 0.82 | 2.38 |  | 0.61 | 0.42 | 5.04 |  | 0.55 | 0.32 | 7.57 |  | 0.76 | 0.45 | 4.40 |  |
|  |  | Fx05toFx01 | 0.72 | 0.49 | 7.72 |  | 0.78 | 0.81 | 2.75 |  | 0.32 | 0.20 | 11.50 |  | 0.52 | 0.29 | 7.18 |  | 0.73 | 0.33 | 4.59 |  |
|  | **Duodenum** | Fx02toFx01 | 0.79 | 0.79 | 5.23 | ✔️ | 0.92 | 0.89 | 2.07 | ✔️ | 0.53 | 0.38 | 14.04 | ✔️ | 0.78 | 0.73 | 15.15 |  | 0.76 | 0.33 | 73.26 | ✔️ |
|  |  | Fx03toFx01 | 0.81 | 0.78 | 2.54 |  | 0.87 | 0.87 | 2.90 |  | 0.52 | 0.30 | 10.70 |  | 0.78 | 0.68 | 4.64 |  | 0.73 | 0.32 | 34.02 |  |
|  |  | Fx04toFx01 | 0.81 | 0.78 | 2.61 |  | 0.91 | 0.88 | 2.89 |  | 0.53 | 0.28 | 9.73 |  | 0.84 | 0.80 | 2.70 |  | 0.76 | 0.33 | 33.68 |  |
|  |  | Fx05toFx01 | 0.81 | 0.78 | 2.58 |  | 0.87 | 0.90 | 2.52 |  | 0.46 | 0.21 | 12.18 |  | 0.81 | 0.76 | 3.25 |  | 0.72 | 0.32 | 13.28 |  |
|  | **Colon** | Fx02toFx01 | 0.86 | 0.72 | 3.91 | ✔️ | 0.58 | 0.24 | 18.47 |  | 0.55 | 0.19 | 16.36 | ✔️ | 0.60 | 0.28 | 16.10 |  | 0.44 | 0.07 | 63.04 |  |
|  |  | Fx03toFx01 | 0.87 | 0.76 | 6.16 |  | 0.46 | 0.17 | 23.35 |  | 0.60 | 0.17 | 13.96 |  | 0.54 | 0.20 | 19.12 |  | 0.45 | 0.06 | 65.06 |  |
|  |  | Fx04toFx01 | 0.86 | 0.73 | 6.53 |  | 0.46 | 0.16 | 24.36 |  | 0.55 | 0.16 | 15.30 |  | 0.51 | 0.19 | 20.52 |  | 0.37 | 0.04 | 64.42 |  |
|  |  | Fx05toFx01 | 0.87 | 0.75 | 5.22 |  | 0.48 | 0.15 | 20.16 |  | 0.60 | 0.19 | 13.07 |  | 0.51 | 0.20 | 19.85 |  | 0.40 | 0.05 | 64.62 |  |

Supplementary Table B.7: (V) Lymph node contour propagation evaluation metrics, DSC (Dice similarity coefficient), SDSC (surface DSC) with 2 mm threshold, and HD95% (Hausdorff distance 95%) for the contours and deformed fraction. G (contour guidance; if checked the contour was used as guidance by the institute. DSC printed in red if < 0.8. Abbreviations; CTV_L_: left clinical target volume, CTV_R_: right clinical target volume.

|  | | | **A** | | | | **C** | | | | **D** | | | | **E** | | | | **F** | | | |
| --- | --- | --- | --- | --- | --- | --- | --- | --- | --- | --- | --- | --- | --- | --- | --- | --- | --- | --- | --- | --- | --- | --- |
|  |  |  | **DSC** | **SDSC** | **HD95% [mm]** | **G** | **DSC** | **SDSC** | **HD95% [mm]** | **G** | **DSC** | **SDSC** | **HD95% [mm]** | **G** | **DSC** | **SDSC** | **HD95% [mm]** | **G** | **DSC** | **SDSC** | **HD95% [mm]** | **G** |
| **(V) Lymph node** | **Bladder** | Fx02toFx01 | 0.99 | 0.99 | 0.62 | ✔️ | 0.96 | 0.95 | 1.43 | ✔️ | 0.69 | 0.14 | 11.01 | ✔️ | 0.77 | 0.63 | 10.64 |  | 0.98 | 0.53 | 1.79 | ✔️ |
|  |  | Fx03toFx01 | 0.99 | 0.99 | 1.04 |  | 0.98 | 0.98 | 1.19 |  | 0.85 | 0.52 | 6.08 |  | 0.96 | 0.91 | 1.90 |  | 0.98 | 0.55 | 3.09 |  |
|  |  | Fx04toFx01 | 0.99 | 1.00 | 1.02 |  | 0.99 | 0.99 | 1.17 |  | 0.84 | 0.43 | 7.11 |  | 0.97 | 0.92 | 1.82 |  | 0.98 | 0.53 | 3.13 |  |
|  |  | Fx05toFx01 | 0.99 | 0.99 | 1.03 |  | 0.98 | 0.98 | 1.22 |  | 0.92 | 0.58 | 3.45 |  | 0.97 | 0.93 | 1.64 |  | 0.98 | 0.54 | 3.01 |  |
|  | **Rectum** | Fx02toFx01 | 0.98 | 1.00 | 0.70 | ✔️ | 0.97 | 0.99 | 1.24 | ✔️ | 0.78 | 0.54 | 6.67 | ✔️ | 0.89 | 0.96 | 1.68 |  | 0.96 | 0.63 | 2.61 | ✔️ |
|  |  | Fx03toFx01 | 0.98 | 1.00 | 0.74 |  | 0.96 | 0.99 | 1.01 |  | 0.76 | 0.50 | 6.68 |  | 0.87 | 0.82 | 2.55 |  | 0.95 | 0.61 | 2.62 |  |
|  |  | Fx04toFx01 | 0.98 | 0.99 | 0.82 |  | 0.97 | 0.99 | 1.26 |  | 0.70 | 0.44 | 8.19 |  | 0.86 | 0.95 | 6.08 |  | 0.95 | 0.60 | 2.74 |  |
|  |  | Fx05toFx01 | 0.98 | 0.99 | 0.79 |  | 0.97 | 0.98 | 1.27 |  | 0.79 | 0.60 | 6.23 |  | 0.89 | 0.97 | 1.27 |  | 0.95 | 0.60 | 2.50 |  |
|  | **Sigmoid** | Fx02toFx01 | 0.76 | 0.88 | 51.75 | ✔️ | 0.64 | 0.62 | 52.19 |  | 0.26 | 0.29 | 55.91 | ✔️ | 0.61 | 0.60 | 54.68 |  | 0.96 | 0.32 | 2.61 |  |
|  |  | Fx03toFx01 | 0.63 | 0.80 | 52.98 |  | 0.40 | 0.62 | 61.45 |  | 0.39 | 0.55 | 58.96 |  | 0.37 | 0.58 | 66.65 |  | 0.95 | 0.40 | 2.62 |  |
|  |  | Fx04toFx01 | 0.76 | 0.88 | 50.16 |  | 0.51 | 0.52 | 51.46 |  | 0.31 | 0.22 | 11.27 |  | 0.55 | 0.56 | 25.83 |  | 0.95 | 0.27 | 2.74 |  |
|  |  | Fx05toFx01 | 0.80 | 0.89 | 22.98 |  | 0.53 | 0.59 | 56.27 |  | 0.52 | 0.50 | 12.43 |  | 0.59 | 0.63 | 52.89 |  | 0.95 | 0.32 | 2.50 |  |
|  | **CTV_L_** | Fx02toFx01 | 0.72 | 1.00 | 1.37 |  | 0.89 | 1.00 | 0.79 | ✔️ | 0.46 | 0.84 | 2.90 |  | 0.58 | 0.95 | 2.22 |  | 0.68 | 0.91 | 1.75 | ✔️ |
|  |  | Fx03toFx01 | 0.40 | 0.92 | 3.44 |  | 0.87 | 1.00 | 0.77 |  | 0.00 | 0.44 | 7.09 |  | 0.27 | 0.89 | 3.67 |  | 0.33 | 0.86 | 4.21 |  |
|  |  | Fx04toFx01 | 0.67 | 1.00 | 2.04 |  | 0.91 | 1.00 | 0.90 |  | 0.00 | 0.41 | 7.27 |  | 0.53 | 0.94 | 2.49 |  | 0.53 | 0.88 | 2.48 |  |
|  |  | Fx05toFx01 | 0.70 | 0.98 | 1.34 |  | 0.55 | 0.99 | 0.94 |  | 0.00 | 0.00 | n.a. |  | 0.69 | 0.96 | 1.27 |  | 0.51 | 0.86 | 2.55 |  |
|  | **CTV_R_** | Fx02toFx01 | 0.82 | 1.00 | 1.01 |  | 0.91 | 1.00 | 0.87 | ✔️ | 0.00 | 0.22 | 6.98 |  | 0.52 | 0.75 | 3.18 |  | 0.75 | 0.90 | 1.90 | ✔️ |
|  |  | Fx03toFx01 | 0.83 | 0.97 | 1.20 |  | 0.87 | 1.00 | 0.88 |  | 0.58 | 0.84 | 2.27 |  | 0.22 | 0.49 | 4.26 |  | 0.74 | 0.92 | 2.46 |  |
|  |  | Fx04toFx01 | 0.82 | 1.00 | 1.15 |  | 0.49 | 1.00 | 1.02 |  | 0.02 | 0.39 | 7.04 |  | 0.31 | 0.50 | 3.91 |  | 0.72 | 0.93 | 1.83 |  |
|  |  | Fx05toFx01 | 0.82 | 0.98 | 1.17 |  | 0.55 | 1.00 | 1.04 |  | 0.00 | 0.00 | n.a. |  | 0.62 | 0.84 | 2.64 |  | 0.69 | 0.94 | 2.40 |  |

Supplementary Table B.8: Summary of the ANOVA for repeated measures with post-hoc test for DSC analysis. Abbreviations CTV: clinical target volume, GTV: gross tumor volume, CTV_HR_: High risk clinical target volume, CTV_IR_: Intermediate risk clinical target volume, CTV_L_: left clinical target volume, CTV_R_: right clinical target volume, A-F are the Institutes, N/A: not applicable, n.s.: not statistically significant.

| Cases & contours | | ANOVA for repeated measures with post-hoc test | | |
| --- | --- | --- | --- | --- |
|  |  | Global hypothesis | Pairwise comparison | |
|  |  | p-Value | Combination of pairs | p-Value |
| Gold  Standard | Bladder | <0.05 | A C  A F  C F  D E | n.s. |
|  |  |  | all other combination pairs | < 0.05 |
|  | Rectum |  | A D  A F  C D  C F  E F | < 0.05 |
|  |  |  | all other combination pairs | n.s. |
|  | Sphincter |  | A F  C F  D F  E F | < 0.05 |
|  |  |  | all other combination pairs | n.s. |
|  | Femur_L_ |  | A D  C D  D E  D F | < 0.05 |
|  |  |  | all other combination pairs | n.s. |
|  | Femur_R_ |  | A D  C D  D E | < 0.05 |
|  |  |  | all other combination pairs | n.s. |
|  | CTV |  | C D  C E  C F | < 0.05 |
|  |  |  | all other combination pairs | n.s. |
|  | GTV |  | C F | < 0.05 |
|  |  |  | all other combination pairs | n.s. |
| (I) Prostate 1 | Bladder | <0.05 | A E | < 0.05 |
|  |  |  | all other combination pairs | n.s. |
|  | Rectum |  | A D  A E  A F | < 0.05 |
|  |  |  | all other combination pairs | n.s. |
|  | Sphincter |  | all combination pairs | n.s |
|  | Femur_L_ |  | A D  C D  D E  D F | < 0.05 |
|  |  |  | all other combination pairs | n.s. |
|  | Femur_R_ |  | A D  C D  D E  D F | < 0.05 |
|  |  |  | all other combination pairs | n.s. |
|  | CTV |  | A D  A E  C F  D E | n.s. |
|  |  |  | all other combination pairs | < 0.05 |
|  | GTV | 0.78 | N/A | |
| (II) Prostate 2 | Bladder | <0.05 | A D  A E  C E  E F | < 0.05 |
|  |  |  | all other combination pairs | n.s. |
|  | Rectum |  | A D  A E  A F  C D | < 0.05 |
|  |  |  | all other combination pairs | n.s. |
|  | Sphincter | 0.90 | N/A | |
|  | Femur_L_ | <0.05 | A D  C D  D E  D F | < 0.05 |
|  |  |  | all other combination pairs | n.s. |
|  | Femur_R_ |  | A D  C D  D E  D F | < 0.05 |
|  |  |  | all other combination pairs | n.s. |
|  | CTV |  | A D  A E  D E | n.s. |
|  |  |  | all other combination pairs | < 0.05 |
|  | GTV | 0.21 | N/A | |
| (III) Cervix | Bladder | <0.05 | A D  C D  D E  D F | < 0.05 |
|  |  |  | all other combination pairs | n.s. |
|  | Rectum |  | A C  A F  C F  E F | n.s. |
|  |  |  | all other combination pairs | < 0.05 |
|  | Sigmoid |  | A C  E F | n.s |
|  |  |  | all other combination pairs | < 0.05 |
|  | CTV_HR_ | 0.70 | N/A | |
|  | CTV_IR_ | 0.99 | N/A | |
|  | GTV | 0.26 | N/A | |
| (IV) Liver | Colon | <0.05 | C D  C E  C F  D E | n.s. |
|  |  |  | all other combination pairs | < 0.05 |
|  | Duodenum |  | A E | n.s. |
|  |  |  | all other combination pairs | < 0.05 |
|  | Kidney_L_ |  | A E  D E  D F | n.s. |
|  |  |  | all other combination pairs | < 0.05 |
|  | Kidney_R_ |  | C E  C F  E F | n.s. |
|  |  |  | all other combination pairs | < 0.05 |
|  | Pancreas |  | A E  A F  E F | n.s. |
|  |  |  | all other combination pairs | < 0.05 |
|  | SmallBowel |  | A C  A D  A E  A F | < 0.05 |
|  |  |  | all other combination pairs | n.s. |
|  | SpinalCord | 0.59 | N/A | |
|  | Spleen | <0.05 | C E  C F  E F | n.s. |
|  |  |  | all other combination pairs | < 0.05 |
|  | Stomach |  | C F | n.s. |
|  |  |  | all other combination pairs | < 0.05 |
|  | Liver |  | C E  C F  E F | n.s. |
|  |  |  | all other combination pairs | < 0.05 |
|  | GTV |  | A C  A F  C F  D E | n.s. |
|  |  |  | all other combination pairs | < 0.05 |
| (V) Lymph node | Bladder | <0.05 | A D  C D  D F | < 0.05 |
|  |  |  | all other combination pairs | n.s. |
|  | Sigmoid |  | A C  A D | < 0.05 |
|  |  |  | all other combination pairs | n.s. |
|  | Rectum |  | A C  A F  C F | n.s. |
|  |  |  | all other combination pairs | < 0.05 |
|  | CTV_L_ |  | A D  C D  D E  D F | < 0.05 |
|  |  |  | all other combination pairs | n.s. |
|  | CTV_R_ |  | A D  A E  C D  D F | < 0.05 |
|  |  |  | all other combination pairs | n.s. |


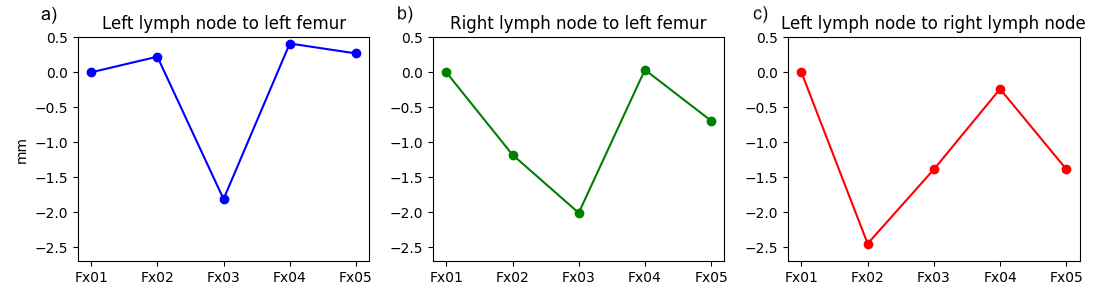


Supplementary Figure B.1: Differences in magnitude vectors a) left lymph node to left femur (mm)

b) right lymph node to left femur (mm) and c) left lymph node to right lymph node (mm) of fractions 2-5 to fraction 1. The magnitude vectors were calculated from the x, y and z coordinates of the vectors a) left lymph node to left femur (mm) b) right lymph node to left femur (mm) and c) left lymph node to right lymph node (mm) which were calculated from the center of mass of the left lymph node, right lymph node, and left femur.

Supplementary Table B.9: Summary of the intraclass correlation coefficient (ICC) and the ANOVA for repeated measures with post-hoc test. Abbreviations CTV: clinical target volume, GTV: gross tumor volume, CTV_HR_: High risk clinical target volume, CTV_IR_: Intermediate risk clinical target volume, CTV_L_: left CTV, CTV_R_: right CTV, A-F are the Institutes, N/A: not applicable, n.s.: not statistically significant.

| Cases & contours | | Reliability | | | ANOVA for repeated measures with post-hoc test | | |
| --- | --- | --- | --- | --- | --- | --- | --- |
|  |  |  |  |  | Global hypothesis | Pairwise comparison | |
|  |  | ICC | 95% Confidence Interval | | p-Value | Combination of pairs | p-Value |
| Gold Standard | CTV | 1.00 | 1.00 | 1.00 | <0.05 | all combination pairs | <0.05 |
|  | Bladder | 1.00 | 1.00 | 1.00 | <0.05 | A        B  A        F  B        F  all other combination pairs | n.s  n.s  n.s  <0.05 |
|  | Rectum | 0.99 | 0.99 | 0.99 | <0.05 | all combination pairs | <0.05 |
| (I) Prostate 1 | CTV | 1.00 | 1.00 | 1.00 | 0.24 | N/A | |
|  | Bladder | 0.99 | 0.99 | 0.99 | <0.05 | all combination pairs | <0.05 |
|  | Rectum | 0.99 | 0.99 | 0.99 | <0.05 | all combination pairs | <0.05 |
| (II) Prostate 2 | CTV | 1.00 | 1.00 | 1.00 | <0.05 | A        C  C        F  all other combination pairs | n.s  n.s  <0.05 |
|  | Bladder | 1.00 | 1.00 | 1.00 | <0.05 | D        E  all other combination pairs | n.s  <0.05 |
|  | Rectum | 0.98 | 0.98 | 0.98 | <0.05 | all pair of combination | <0.05 |
| (III) Cervix | GTV | 1.00 | 1.00 | 1.00 | <0.05 | D        F  all other combination pairs | n.s  <0.05 |
|  | CTV_HR_ | 1.00 | 1.00 | 1.00 | <0.05 | all pair of combination | <0.05 |
|  | CTV_IR_ | 1.00 | 1.00 | 1.00 | <0.05 | all pair of combination | <0.05 |
|  | Bladder | 1.00 | 1.00 | 1.00 | <0.05 | B        D  all other combination pairs | n.s  <0.05 |
|  | Rectum | 1.00 | 1.00 | 1.00 | <0.05 | A        C  all other combination pairs | n.s  <0.05 |
|  | Sigmoid | 1.00 | 1.00 | 1.00 | <0.05 | A        E  C        E  all other combination pairs | n.s  n.s  <0.05 |
| (IV) Liver | GTV | 0.92 | 0.92 | 0.92 | 0.33 | N/A | |
|  | Colon | 1.00 | 1.00 | 1.00 | <0.05 | all pair of combination | <0.05 |
|  | Duodenum | 1.00 | 1.00 | 1.00 | <0.05 | all pair of combination | <0.05 |
|  | Small bowel | 0.99 | 0.99 | 0.99 | <0.05 | all pair of combination | <0.05 |
|  | Spinal cord | 0.99 | 0.99 | 0.99 | 0.22 | N/A | |
|  | Liver | 1.00 | 1.00 | 1.00 | <0.05 | A        D  all other combination pairs | n.s  <0.05 |
| (V) Lymph node | Bladder | 1.00 | 1.00 | 1.00 | 0.05 | N/A | |
|  | Rectum | 1.00 | 1.00 | 1.00 | <0.05 | A        F  all other combination pairs | n.s  <0.05 |
|  | Sigmoid | 1.00 | 1.00 | 1.00 | <0.05 | A        E  A        F   E        F  all other combination pairs | n.s  n.s  n.s  <0.05 |
|  | CTV_L_ | 0.84 | 0.84 | 0.85 | <0.05 | all pair of combination | <0.05 |
|  | CTV_R_ | 0.72 | 0.71 | 0.73 | <0.05 | A        E  all other combination pairs | n.s  <0.05 |


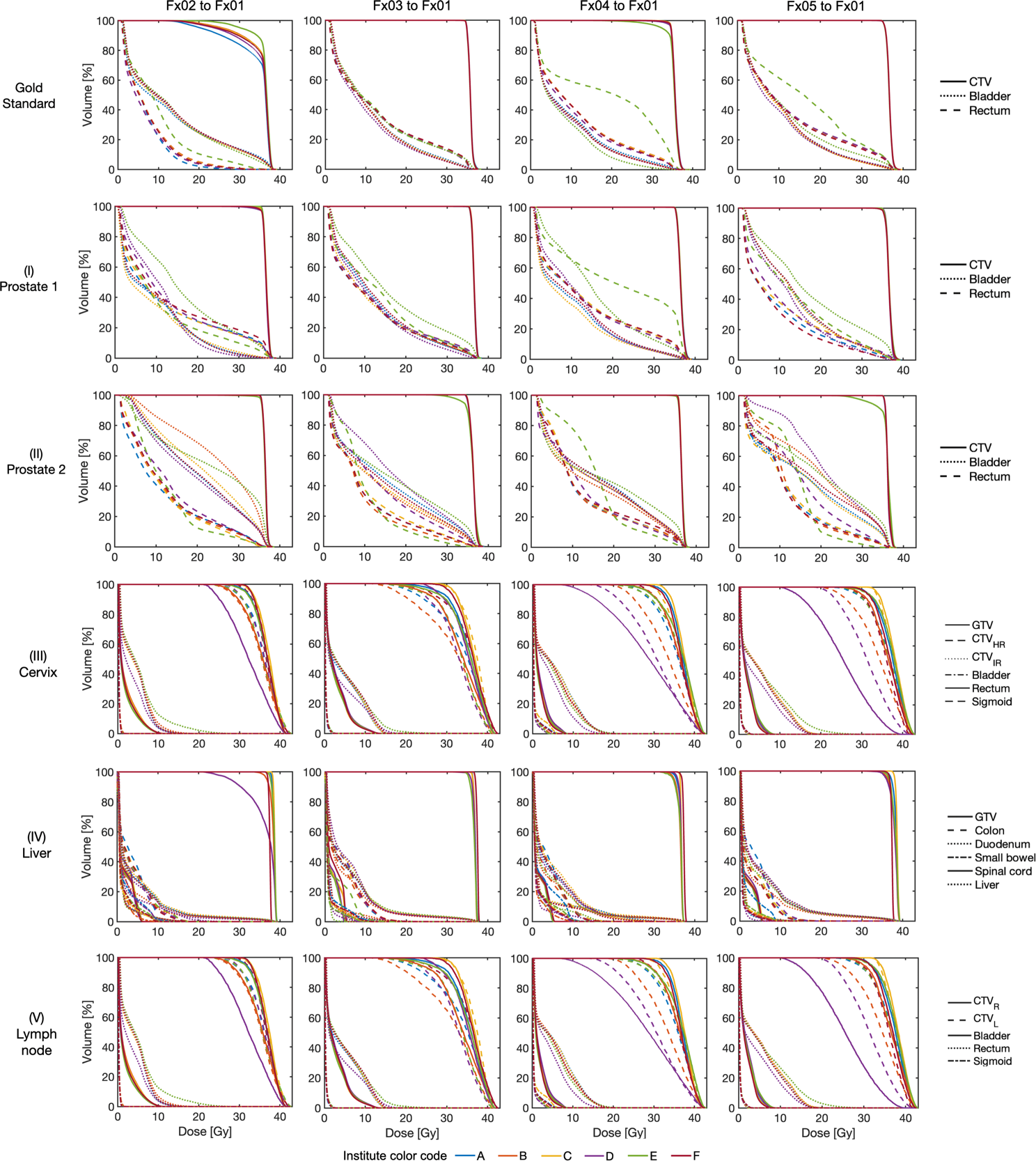


Supplementary Figure B.2: Gold standard and clinical cases resulted DHVs of the deformed dose for fraction two (Fx02) till fraction five (Fx05) to fraction one (Fx01) by the use of offline re-contoured structure set of fraction one (Fx01). The colors illustrate the different institutes (A-F) and the line type illustrates the clinically relevant contours. Note: Institute B did not provide mapped dose files for cervix and prostate 1. Abbreviations; CTV: clinical target volume, GTV: gross tumor volume, CTV_HR_: High risk clinical target volume, CTV_IR_: Intermediate risk clinical target volume, CTV_L_: left CTV, CTV_R_: right CTV.


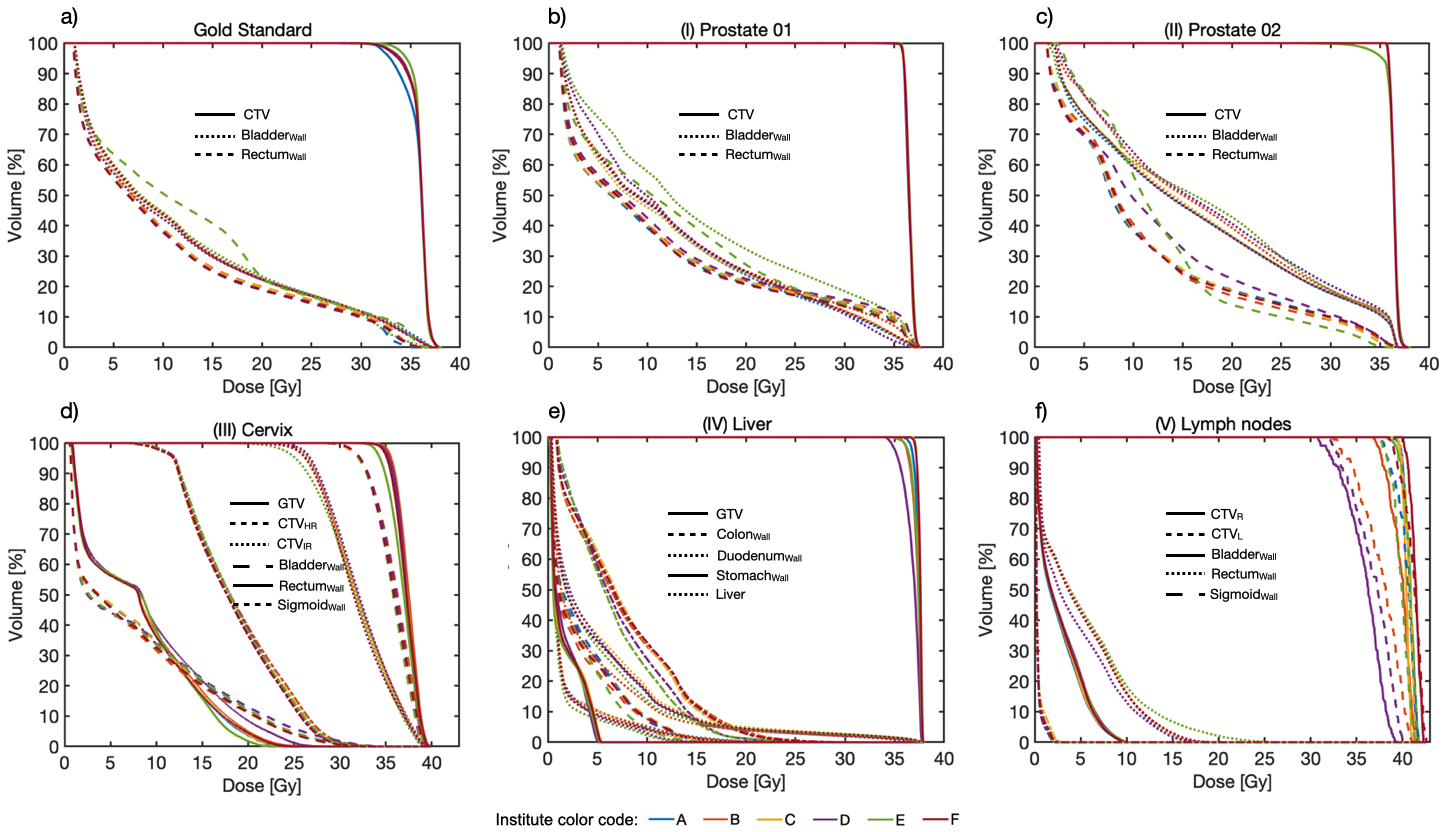


Supplementary Figure B.3: Results of the accumulated DVH per tumor volume and wall contours and institutes; for the cases gold standard (a), (I) prostate 1 (b), (II) prostate 2 (c), (III) cervix (d), (IV) liver (e) and (V) lymph node (f). Wall contours for rectum, colon, and duodenum, likewise for bladder and stomach were created using negative margins of 3 and 4 mm. The institutes (A-F) are presented in different colors, the case-related contours are presented in different line styles. Contours of fraction one were used for the respective DVH calculation.

Supplementary Table B.10: Clinical dosimetric criteria (CDC) results of deformable dose accumulation (DDA) of the institutes for the gold standard (GS) and the clinical cases; (I) prostate 1, (II) prostate 2, (III) cervix, (IV) liver, and (V) lymph nodes. Wall contours for rectum, colon, and duodenum, likewise for bladder and stomach were created using negative margins of 3 and 4 mm. The respective CDC for the individual institutes DDA were calculated by Monaco by the use of offline re-contoured wall structure set of fraction one (Fx01).

| Case | Clinical dosimetric criteria (CDC) | | | | Accumulated | | | | | | | |
| --- | --- | --- | --- | --- | --- | --- | --- | --- | --- | --- | --- | --- |
|  |  |  |  |  | A | B | C | D | E | F | Mean | Std |
| Gold Standard | Bladder_Wall_ | D5cm^3^ | < 37.0 | [Gy] | 33.0 | 32.7 | 32.8 | 32.4 | 31.4 | 32.6 | 32.5 | 0.6 |
|  |  | V28.0Gy | < 15.0 | [%] | 14.1 | 14.0 | 14.0 | 13.6 | 13.9 | 13.9 | 13.9 | 0.2 |
|  |  | V32.0Gy | < 20.0 | [%] | 9.5 | 9.3 | 9.3 | 8.8 | 6.4 | 9.1 | 8.7 | 1.2 |
|  | Rectum_Wall_ | D1cm^3^ | ≤  38.0 | [Gy] | 33.1 | 34.3 | 34.3 | 34.1 | 35.4 | 34.0 | 34.2 | 0.7 |
|  |  | V28.0Gy | ≤ 15.0 | [%] | 12.0 | 12.9 | 13.1 | 11.8 | 13.7 | 12.0 | 12.6 | 0.8 |
|  |  | V32.0Gy | ≤  20.0 | [%] | 6.7 | 8.2 | 8.4 | 7.4 | 9.9 | 7.3 | 8.0 | 1.1 |
| (I) Prostate1 | Bladder_Wall_ | D5cm^3^ | < 37.0 | [Gy] | 37.1 | 33.9 | 32.3 | 31.2 | 35.6 | 30.1 | 33.3 | 2.7 |
|  |  | V28.0Gy | < 15.0 | [%] | 13.6 | 15.8 | 14.4 | 14.3 | 21.1 | 5.2 | 14.1 | 5.1 |
|  |  | V32.0Gy | < 20.0 | [%] | 8.5 | 11.3 | 9.5 | 7.3 | 15.7 | 2.5 | 9.1 | 4.4 |
|  | Rectum_Wall_ | D1cm^3^ | ≤  38.0 | [Gy] | 36.5 | 36.4 | 36.6 | 36.8 | 36.6 | 36.4 | 36.6 | 0.2 |
|  |  | V28.0Gy | ≤ 15.0 | [%] | 15.4 | 16.3 | 16.0 | 16.9 | 16.0 | 15.0 | 15.9 | 0.7 |
| (II) Prostate2 | Bladder_Wall_ | D5cm^3^ | < 37.0 | [Gy] | 35.8 | 36.0 | 35.9 | 36.1 | 36.1 | 35.8 | 35.9 | 0.1 |
|  |  | V28.0Gy | < 15.0 | [%] | 20.7 | 22.0 | 20.7 | 23.9 | 22.6 | 20.8 | 21.8 | 1.3 |
|  |  | V32.0Gy | < 20.0 | [%] | 15.2 | 17.0 | 15.4 | 17.3 | 15.8 | 15.2 | 16.0 | 0.9 |
|  | Rectum_Wall_ | D1cm^3^ | ≤  38.0 | [Gy] | 34.9 | 34.9 | 34.3 | 35.1 | 33.1 | 30.1 | 33.7 | 1.9 |
|  |  | V28.0Gy | ≤ 15.0 | [%] | 11.8 | 10.5 | 11.1 | 13.2 | 7.7 | 11.6 | 11.0 | 1.8 |
|  |  | V32.0Gy | ≤  20.0 | [%] | 7.8 | 7.3 | 6.8 | 8.5 | 4.4 | 8.1 | 7.2 | 1.5 |
| (III)  Cervix | Bladder_Wall_ | D2cm^3^ | < 27.5 | [Gy] | 26.5 | 27.0 | 26.5 | 26.3 | 26.6 | 26.2 | 26.5 | 0.3 |
|  | Rectum_Wall_ | D2cm^3^ | < 21.5 | [Gy] | 17.3 | 18.0 | 17.6 | 19.7 | 16.6 | 17.5 | 17.8 | 1.0 |
|  | Sigmoid_Wall_ | D2cm^3^ | < 21.5 | [Gy] | 21.7 | 21.3 | 21.3 | 22.6 | 22.0 | 21.6 | 21.8 | 0.5 |
| (IV)  Liver | Colon_Wall_ | Dmax | < 32.0 | [Gy] | 22.7 | 23.3 | 22.5 | 19.3 | 17.3 | 22.6 | 21.3 | 2.4 |
|  | Duodenum_Wall_ | Dmax | < 30.0 | [Gy] | 24.0 | 21.9 | 22.3 | 17.2 | 16.5 | 22.9 | 20.8 | 3.2 |
|  | Stomach_Wall_ | Dmax | < 30.0 | [Gy] | 29.7 | 30.1 | 30.3 | 26.9 | 28.0 | 30.4 | 29.2 | 1.4 |
| (V) Lymph node | Bladder_Wall_ | D0.5cm^3^ | < 32.0 | [Gy] | 9.0 | 9.1 | 9.1 | 9.2 | 9.3 | 9.1 | 9.1 | 0.1 |
|  | Rectum_Wall_ | D0.5cm^3^ | < 32.0 | [Gy] | 14.5 | 15.0 | 14.8 | 14.3 | 19.2 | 15.2 | 15.5 | 1.8 |
|  | Sigmoid_Wall_ | D0.5cm^3^ | < 32.0 | [Gy] | 1.7 | 1.5 | 1.9 | 1.3 | 1.5 | 1.6 | 1.6 | 0.2 |

References

[1] Paulson ES, Ahunbay E, Chen X, Mickevicius NJ, Chen G-P, Schultz C, et al. 4D-MRI driven MR-guided online adaptive radiotherapy for abdominal stereotactic body radiation therapy on a high field MR-Linac: Implementation and initial clinical experience. Clin Transl Radiat Oncol 2020;23:72–9. https://doi.org/10.1016/j.ctro.2020.05.002.

[2] Han X, Hibbard LS, Willcut V. An Efficient Inverse-Consistent Diffeomorphic Image Registration Method for Prostate Adaptive Radiotherapy. In: Madabhushi A, Dowling J, Yan P, Fenster A, Abolmaesumi P, Hata N, editors. Prostate Cancer Imaging Comput.-Aided Diagn. Progn. Interv., Berlin, Heidelberg: Springer; 2010, p. 34–41. https://doi.org/10.1007/978-3-642-15989-3_5.

[3] Fedorov A, Beichel R, Kalpathy-Cramer J, Finet J, Fillion-Robin J-C, Pujol S, et al. 3D Slicer as an image computing platform for the Quantitative Imaging Network. Magn Reson Imaging 2012;30:1323–41. https://doi.org/10.1016/j.mri.2012.05.001.

[4] Wang H, Dong L, Lii MF, Lee AL, de Crevoisier R, Mohan R, et al. Implementation and validation of a three-dimensional deformable registration algorithm for targeted prostate cancer radiotherapy. Int J Radiat Oncol 2005;61:725–35. https://doi.org/10.1016/j.ijrobp.2004.07.677.

[5] Thirion J-P. Image matching as a diffusion process: an analogy with Maxwell’s demons. Med Image Anal 1998;2:243–60. https://doi.org/10.1016/S1361-8415(98)80022-4.

[6] Zhang Y, Zhang L, Court LE, Balter P, Dong L, Yang J. Tissue-specific deformable image registration using a spatial-contextual filter. Comput Med Imaging Graph Off J Comput Med Imaging Soc 2021;88:101849. https://doi.org/10.1016/j.compmedimag.2020.101849.

[7] Ger RB, Yang J, Ding Y, Jacobsen MC, Fuller CD, Howell RM, et al. Accuracy of deformable image registration on magnetic resonance images in digital and physical phantoms. Med Phys 2017;44:5153–61. https://doi.org/10.1002/mp.12406.

[8] Lakomy DS, Yang J, Vedam S, Wang J, Lee B, Sobremonte A, et al. Clinical Implementation and Initial Experience With a 1.5 Tesla MR-Linac for MR-Guided Radiation Therapy for Gynecologic Cancer: An R-IDEAL Stage 1 and 2a First in Humans Feasibility Study of New Technology Implementation. Pract Radiat Oncol 2022;12:e296–305. https://doi.org/10.1016/j.prro.2022.03.002.

[9] Lim SY, Tran A, Tran ANK, Sobremonte A, Fuller CD, Simmons L, et al. Dose accumulation of daily adaptive plans to decide optimal plan adaptation strategy for head-and-neck patients treated with MR-Linac. Med Dosim Off J Am Assoc Med Dosim 2022;47:103. https://doi.org/10.1016/j.meddos.2021.08.005.

[10] Yang J, Vedam S, Lee B, Castillo P, Sobremonte A, Hughes N, et al. Online adaptive planning for prostate stereotactic body radiotherapy using a 1.5 Tesla magnetic resonance imaging-guided linear accelerator. Phys Imaging Radiat Oncol 2021;17:20–4. https://doi.org/10.1016/j.phro.2020.12.001.

[11] Haber E, Modersitzki J. Intensity Gradient Based Registration and Fusion of Multi-modal Images. In: Larsen R, Nielsen M, Sporring J, editors. Med. Image Comput. Comput.-Assist. Interv. – MICCAI 2006, Berlin, Heidelberg: Springer; 2006, p. 726–33. https://doi.org/10.1007/11866763_89.

[12] Bosma LS, Ries M, Denis de Senneville B, Raaymakers BW, Zachiu C. Integration of operator-validated contours in deformable image registration for dose accumulation in radiotherapy. Phys Imaging Radiat Oncol 2023;27:100483. https://doi.org/10.1016/j.phro.2023.100483.

[13] Li HS, Zhong H, Kim J, Glide-Hurst C, Gulam M, Nurushev TS, et al. Direct dose mapping versus energy/mass transfer mapping for 4D dose accumulation: fundamental differences and dosimetric consequences. Phys Med Biol 2014;59:173–88. https://doi.org/10.1088/0031-9155/59/1/173.

[14] Brock KK, Mutic S, McNutt TR, Li H, Kessler ML. Use of image registration and fusion algorithms and techniques in radiotherapy: Report of the AAPM Radiation Therapy Committee Task Group No. 132. Med Phys 2017;44:e43–76. https://doi.org/10.1002/mp.12256.

[15] Huttenlocher DP, Klanderman GA, Rucklidge WJ. Comparing images using the Hausdorff distance. IEEE Trans Pattern Anal Mach Intell 1993;15:850–63. https://doi.org/10.1109/34.232073.

[16] Rong Y, Rosu-Bubulac M, Benedict SH, Cui Y, Ruo R, Connell T, et al. Rigid and Deformable Image Registration for Radiation Therapy: A Self-Study Evaluation Guide for NRG Oncology Clinical Trial Participation. Pract Radiat Oncol 2021;11:282–98. https://doi.org/10.1016/j.prro.2021.02.007.
